# Disease-microenvironment modulation by bare- or engineered-exosome for rheumatoid arthritis treatment

**DOI:** 10.1186/s40824-023-00418-2

**Published:** 2023-08-28

**Authors:** Eun Sook Lee, Hyewon Ko, Chan Ho Kim, Hyun-Chul Kim, Seong-Kyoon Choi, Sang Won Jeong, Se-Guen Lee, Sung-Jun Lee, Hee-Kyung Na, Jae Hyung Park, Jung Min Shin

**Affiliations:** 1 Safety Measurement Institute, Korea Research Institute of Standards and Science (KRISS), 267 Gajeong-Ro, Yuseong-Gu, Daejeon, 34113 Republic of Korea; 2 Bionanotechnology Research Center, Korea Research Institute of Bioscience & Biotechnology (KRIBB), Yuseong-Gu, Daejeon, 34141 Republic of Korea; 3 School of Chemical Engineering, Sungkyunkwan University, Suwon, 16419 Republic of Korea; 4 Division of Biotechnology, Convergence Research Institute, DGIST, 333 Techno Jungang-Daero, Daegu, 42988 Republic of Korea; 5 Present Address: Department of Polymer Science and Engineering, Korea National University of Transportation, Chungju, 27469 Republic of Korea

**Keywords:** Exosome, Rheumatoid arthritis, Disease microenvironment, Engineered exosome

## Abstract

**Background:**

Exosomes are extracellular vesicles secreted by eukaryotic cells and have been extensively studied for their surface markers and internal cargo with unique functions. A deeper understanding of exosomes has allowed their application in various research areas, particularly in diagnostics and therapy.

**Main body:**

Exosomes have great potential as biomarkers and delivery vehicles for encapsulating therapeutic cargo. However, the limitations of bare exosomes, such as rapid phagocytic clearance and non-specific biodistribution after injection, pose significant challenges to their application as drug delivery systems. This review focuses on exosome-based drug delivery for treating rheumatoid arthritis, emphasizing pre/post-engineering approaches to overcome these challenges.

**Conclusion:**

This review will serve as an essential resource for future studies to develop novel exosome-based therapeutic approaches for rheumatoid arthritis. Overall, the review highlights the potential of exosomes as a promising therapeutic approach for rheumatoid arthritis treatment.

**Graphical Abstract:**

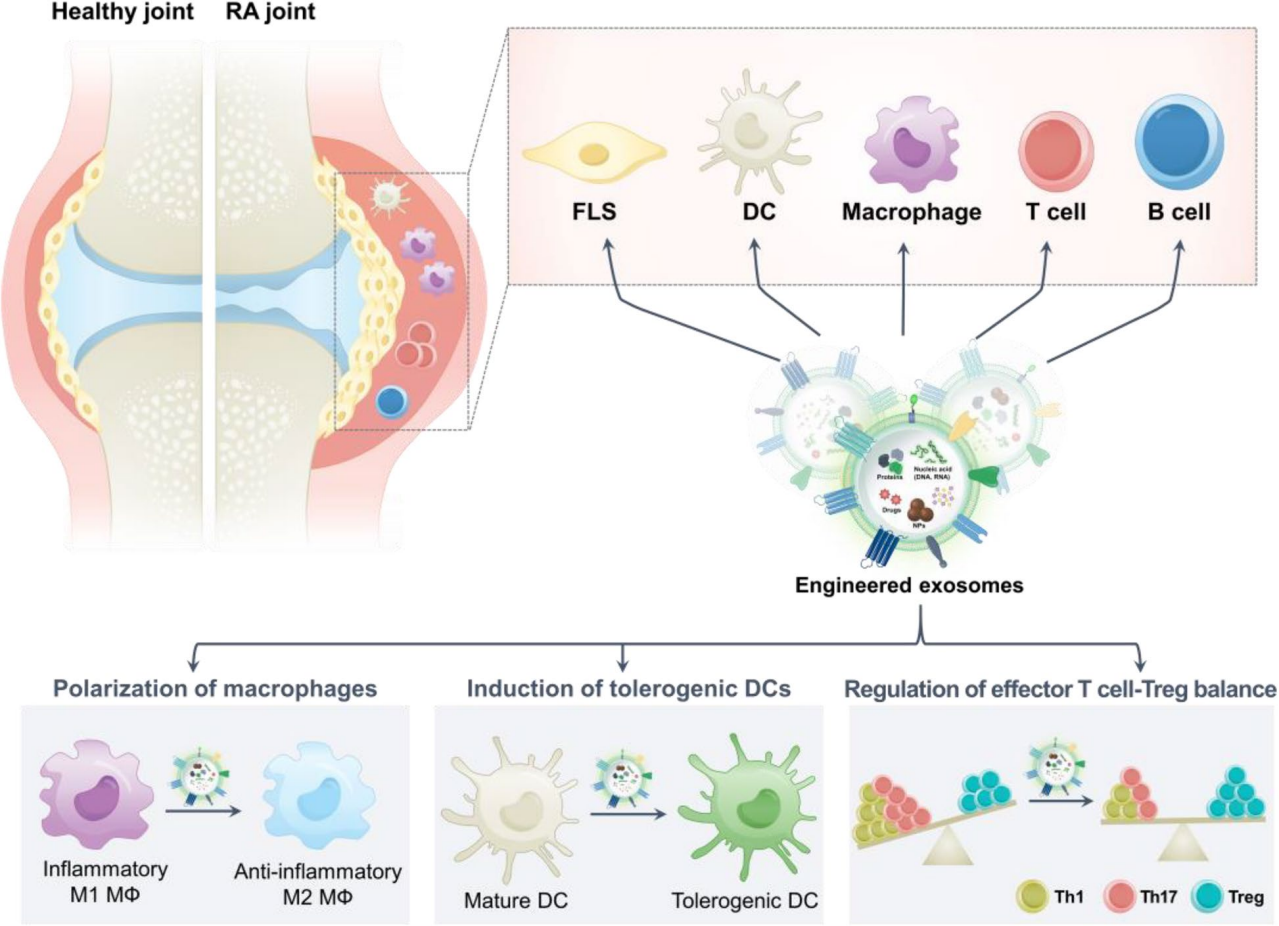

## Background

Exosomes are extracellular vesicles of 30–100 nm diameter and are secreted by most eukaryotic cells [1]. They are secreted from various types of cells and contribute to homeostasis by participating in intercellular interactions [2–4]. Conventionally, exosome research has focused on surface markers or their internally encapsulated cargo. CD9 and CD63 are verified exosome-specific markers [5]. In addition, the components of the internal cargo (lipids, nucleic acids, metabolites) have been characterized [5–7]. A deeper understanding of exosomes furthers understanding of various physiological and pathological phenomena. It provides an opportunity to focus on yet-to-be-conquered diseases and disorders with new perspectives.

An advanced understanding of exosome function has promoted its application in diverse research areas [8], especially in diagnosing and treating diseases [9, 10]. Consequently, efforts are being made to identify exosomes that can function as reliable biomarkers. For example, exosomal PD-L1 from cancer cells can be used as a biomarker to predict the therapeutic effect of immune checkpoint inhibitors [11]. In addition, exosomes have been developed as a delivery vehicle encapsulated with therapeutic cargo [12, 13]. Several studies have attempted to verify the efficacy of exosomes for intractable diseases such as rheumatoid arthritis, cancer, and stroke [14–16] and verified that exosome has excellent potential as drug delivery vehicles. Although bare exosomes have been explored for their potential clinical application, their rapid phagocytic clearance and non-specific biodistribution after injection pose considerable limitations to their application as drug delivery systems. To overcome these limitations, pre- or post-engineering strategies may have exciting implications for developing novel exosome delivery vehicles.

Rheumatoid arthritis (RA) is an autoimmune disease involving the irreversible destruction of joints. Treatments such as nonsteroidal anti-inflammatory drugs (NSAIDs), disease-modifying anti-rheumatic drugs (DMARDs), and tumor necrosis factor-α (TNF-α) inhibitors alleviate RA symptoms and disease progression. However, RA cannot be cured with present therapeutic modalities. Because various immune cells and abnormal immune responses are involved in RA pathogenesis, appropriate immunomodulation may facilitate a more effective RA treatment. Since exosomes can function as effective regulators of immune responses, they may have implications for better RA treatment.

In this review, we provide comprehensively discuss the potential of exosomes as drug delivery vehicles for treating RA, and provide an overview of pre/post-engineered exosomes that can overcome the limitations of bare exosomes.

## Rheumatoid arthritis

RA is a chronic disease that causes severe pain, swelling, stiffness, and functional loss of joints [17–19]. Although the exact pathogenesis of this disease is unknown, RA onset is reportedly caused by various risk factors, including genetic, epigenetic, dust inhalation, microbiota, and lifestyle [20–22]. Immune regulatory factors related to nuclear factor kappa B (NF-κB) stimulation, activation, and functional differentiation of T cells are believed to be closely related to RA. Human leukocyte antigen (HLA)-DBR1 reportedly has the most robust relation with RA among genetic factors. The effects of environmental factors are apparent when investigated at the genetic level. For example, smoking and silica inhalation significantly increase the incidence rate of RA in individuals with susceptibility to HLA-DR4 alleles.

**Fig. 1:**
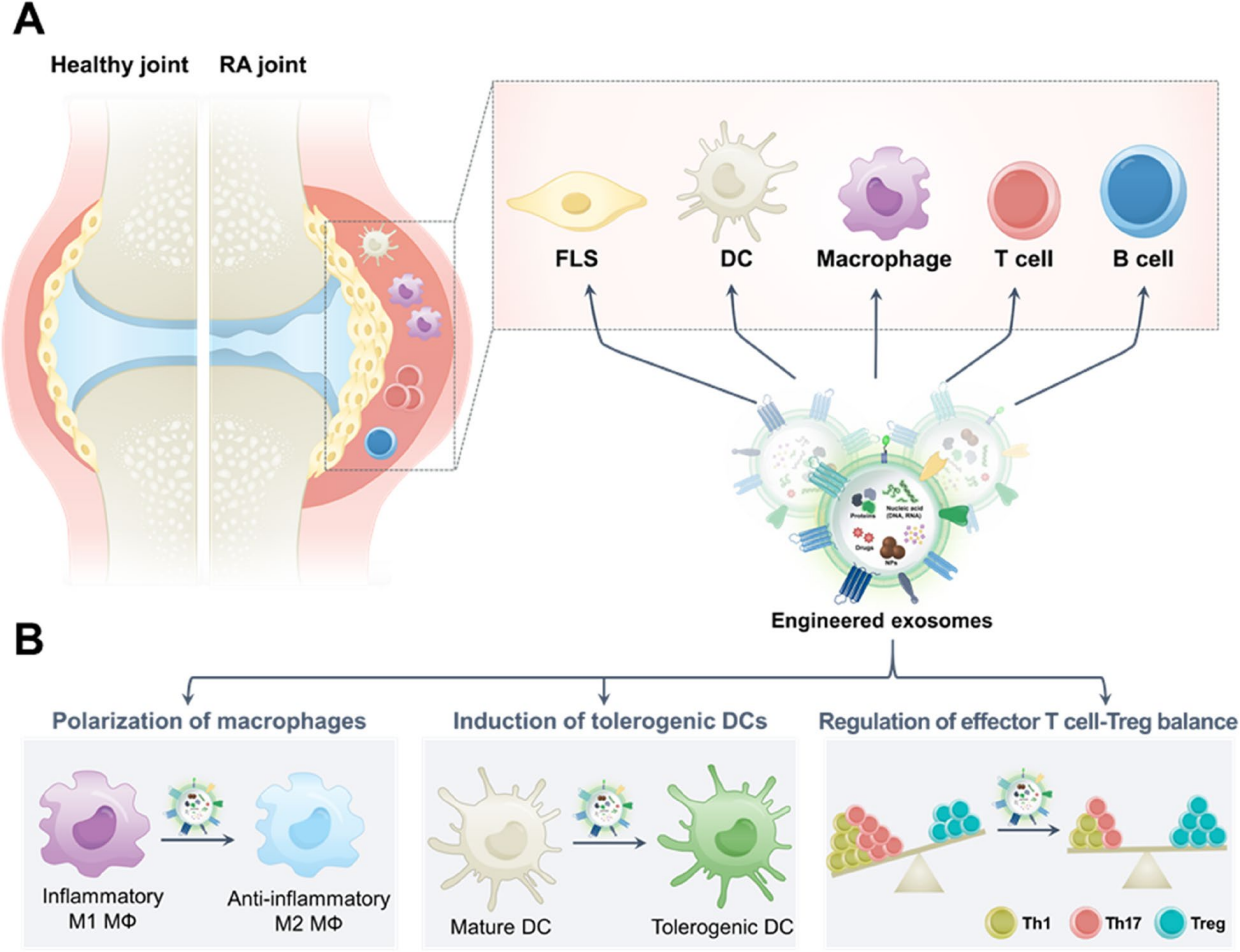
**A** The modulation of various kinds of cells in rheumatoid arthritis-microenvironment by engineered exosomes. **B** Immunosuppression effects on macrophages, dendritic cells (DCs), and T cells. FLS, fibroblast-like synoviocytes; Th1, T-helper 1 cells; Th17, T-helper 17 cells; Treg, Regulatory T cells

Although RA is a systemic autoimmune disease, symptoms usually occur with severe joint inflammation [23]. Thus, targeting the inflammatory disease microenvironment and cell populations is crucial for treating RA [24]. In synovial tissue, highly proliferative immune cells, vascular endothelial cells, and fibroblast-like synoviocytes (FLSs) establish an inflammatory microenvironment and release various factors such as TNF-α, IL-1, IL-6, matrix metalloproteinases (MMPs), and autoantibodies [25–29]. These components facilitate the development of hyperinflammatory microenvironment. In addition, systemic impaired immune functions and an imbalanced proportion of immune cells, such as in the helper T cell and regulatory T cell ratio, further aggravates RA pathology. The cell types closely related to RA pathogenesis include FLSs, macrophages, B cells, T cells, and dendritic cells (DCs) (Fig. 1).

FLS, a significant producer of inflammatory cytokines at the synovial site, is primarily responsible for RA initiation [30, 31]. FLSs constitute a 2–3-layer network in normal synovium. However, in the RA pathological condition, an inflammatory response is induced by continuous FLS proliferation and immune cell accumulation, which causes synovial hyperplasia [32]. Activated FLS converts its network to a pannus-like multi-layer structure in RA lesions. This structure can be identified as a distinct synovium lining hypertrophic lesion [33]. During RA progression, MMP-13 overexpression mediates the destruction of bone and cartilage structures. Multiple studies have reported that MMP-13 production is increased in cytokine-stimulated FLSs [34, 35]. Activated FLSs act as effector cells that induce the activation of inflammatory T cells by expressing IL-7 and IL-15, which possess functional similarities with IL-2 [36, 37]. However, synovial macrophages secrete only IL-15, whereas synovial T cells do not secrete either IL-7 or IL-15. In addition, FLS secrete TNF-α similar to other synovial cells such as lymphocytes, macrophages, and endothelial cells [38]. Therefore, cytokines secreted by synovial immune cells and the distinct cytokine profile of FLS contribute to the RA microenvironment.

Activated macrophages secrete IL-1, IL-6, and TNF-α, and they can be induced by various cell subsets, such as FLSs, monocytes, and helper T cells [39]. The cytokines present in the synovial fluid induce severe inflammation in RA [40, 41]. Initial erosion of RA tissue gives way to synovial hyperplasia [42, 43]. Activated macrophages predominantly accumulate at the borderline of pannus-like multi-layer structure and cartilage along with FLSs [33]. In particular, TNF-α and IL-1 induce FLS activation, producing tissue-destructive MMP [44]. TNF-α inhibition by antibodies leads to a considerable reduction in IL-1, IL-6, IL-8, and GM-CSF. Therefore, TNF-α blockade is an excellent therapeutic strategy to suppress inflammation in RA patients.

CD4^+^ T cells are considered central cellular mediators involved in RA pathogenesis [45]. Among CD4^+^ T cells, helper T cells and regulatory T cells are significantly related to RA. Helper T cells, characterized by CD4 expression on the cell surface, promote inflammation by activating osteoclasts, FLSs, chondrocytes, and immune cells via various inflammatory cytokines. For example, helper T cells secrete IL-17 to activate osteoclast, which causes bone destruction [46]. Similarly, IFN-γ contributes to macrophage polarization to inflammatory M1 phenotype [47]. In addition, cell surface signaling by membrane proteins such as CD69 and CD11 induces the secretion of MMP and other effector molecules by FLS and chondrocytes, which in turn promotes inflammatory responses and causes joint erosion [23]. Another subset of CD4^+^ T cells is regulatory T cells (Tregs), CD4^+^CD25^+^FOXP3^+^ cells [48]. Unlike helper T cells, Tregs are considered effective suppressors of RA [49]. Although many Treg cells are present in the synovial fluid of RA patients, they are functionally deficient. This suggests an immune suppressive role of ‘normal’ Tregs in RA [50]. Thus, synovial Tregs in RA patients often exhibit reduced regulatory capacity, which might be attributed to the severe inflammatory condition of the RA microenvironment [51].

DCs are one of the most professional antigen-presenting cells (APCs) [52]. DCs prime T cells by presenting antigenic peptides on their surface complexed with MHC [53]. Along with MHC class II, co-stimulatory molecules such as CD80 and CD86 facilitate interaction and priming of T cells [54]. Auto-reactive helper T cells primed by DCs contribute to the inflammatory microenvironment by secreting pro-inflammatory cytokines, such as IL-12 and IL-23 [55].

Like other immune cells, B cells are also abnormally increased in RA [56]. During RA initiation, B cells produce autoantigen, TNF-α, and IL-6 [57]. Among them, IL-6 contributes to forming an inflammatory microenvironment in the synovial membrane by promoting monocyte differentiation. In addition, B cells act as APCs in RA to induce helper T cell activation by presenting autoantigens to CD4^+^ T cells [58]. As the disease progress, B cells infiltrate synovial tissue and induce a sustained and destructive autoimmune response.

The synovial microenvironment facilitates optimal antigen processing and presentation by antigen-presenting cells for T cell activation [59]. Studies have revealed that the interaction of activated T cells with monocytes induces the secretion of various inflammatory cytokines and chemokines [60–62]. Hence, the interaction of immune cells and inflammatory factors in the inflamed synovial microenvironment plays a pivotal role in RA pathogenesis and progression.

## Exosome

### Biogenesis of exosomes

Exosomes are nano-sized extracellular vesicles secreted by most eukaryotic cells. Unlike other small extracellular vesicles such as apoptotic bodies and microvesicles, exosomes are primarily generated via cell membrane fusion of exosome-containing endosomes. Exosomes typically have a hydrodynamic size of 30–150 nm in diameter, which can be characterized via nanoparticle trafficking analysis method. The physicochemical properties of exosomes can be characterized through isolation from conditioned cell culture media using ultracentrifugation, size exclusion chromatography, or immunoaffinity-based isolation kits [63]. Exosomes express CD9 and CD63 as surface markers and encapsulate exosome biogenesis-related factors, such as TSG101, ALIX, and Rab27a.

Various studies have recently studied exosome biogenesis, and the accumulated research results provide evidence about exosome biogenesis [1, 64, 65]. Exosomes are generated via direct inward budding of the phospholipid bilayer within the MVB derived from the endosomes and then secreted out of the cell [64]. Exosome secretion occurs through an endosomal sorting complex responsible for transport (ESCRT) protein-dependent or ESCRT-independent mechanism. ESCRT-independent exosome biogenesis showed that sphingolipid ceramide is crucial [66]. This study has revealed that the inhibition of neutral sphingomyelinases reduces the number of exosomes released. In addition, cells can produce CD63-positive MVEs regardless of the depletion of the four subunits of ESCRT [67]. However, several ESCRT proteins are involved in exosome formation and induce ESCRT-dependent exosome biogenesis. For example, the reticulocyte transferrin receptor reportedly interacts with ESCRT accessory protein Alix during exosome formation [68]. Subsequently, Alix contributes to exosome biogenesis and intraluminal vesicle formation by recruiting ESCRT-III complex protein [69].

Various mechanisms determine the contents of the cargo inside the exosome, and as a representative example, proteins such as tetraspanin participate in protein loading in the exosome. Tetraspanin-rich microdomains contribute to the differentiation of receptors and signaling molecules within the exosome plasma membrane [70]. In addition, tetraspanins such as CD9 and CD81 are known to form microdomains and bind to specific proteins; this function serves as a sorting mechanism during the generation of exosomes [70, 71]. Rab27 is an essential protein involved in exosome secretion after biogenesis. Rab27 silencing reduces exosome secretion without changing exosome cargo protein composition [72]. Conversely, inhibiting the proteasomal degradation of Rab27 tends to increase exosome secretion [73].

Various cargo molecules, including DNAs, RNAs, and lipids, are present inside the exosome [74]. Cargos are considered to play an essential role in intercellular communication. In contrast to the previous assumption that exosomes are just cell debris, recent studies have revealed that they are primarily involved in normal biological processes [75]. The exosomes mediate local communication around the recipient cell, and they circulate along the blood vessel after being secreted and accumulate at the target site, acting as mediators in cell-to-cell interactions [76, 77]. Exosomes delivered to the recipient cell enter the recipient cell through fusion, receptor-mediated uptake, and internalization for cell-to-cell communication. A previous study reported that mRNA could be encapsulated in the exosome of the parent cell and translated after being taken up by the recipient cell [7]. Studies on the development of delivery systems have explored the applicability of exosomes to treat diseases [78]. Previous exosome studies reveal that inhibiting exosome biogenesis induces cell apoptosis [64]. Many studies have revealed that exosomes play a crucial role in the progression of intractable diseases, and efforts to understand the exosomes related to the diseases are continuing.

In RA, exosomes significantly contribute to the inflammatory environment of the joints. Numerous exosomes exist in various biological fluids, including synovial fluid [79]. These exosomes are secreted from multiple cell populations such as FLSs, T cells, and B cells [80–83]. They trigger inflammation by delivering damage-associated molecular patterns [84]. In addition, the exosome’s cytokines, lipids, and microRNAs aggravate RA’s inflammation [85]. Exosomes also play critical roles in disease progression in various chronic diseases. For example, cancer exosomes deliver oncogenic cargos, which makes tumors a favorable microenvironment. They also overexpress PD-L1 on their surface, thereby resisting immune checkpoint inhibitors. Disease progression can be delayed, and the therapeutic effect of conventional therapies can be improved by targeting disease-favorable exosomes. Therefore, exosomes are potential biomarkers in chronic diseases and have the potential to develop therapeutic approaches [86].

### Engineering

**Fig. 2:**
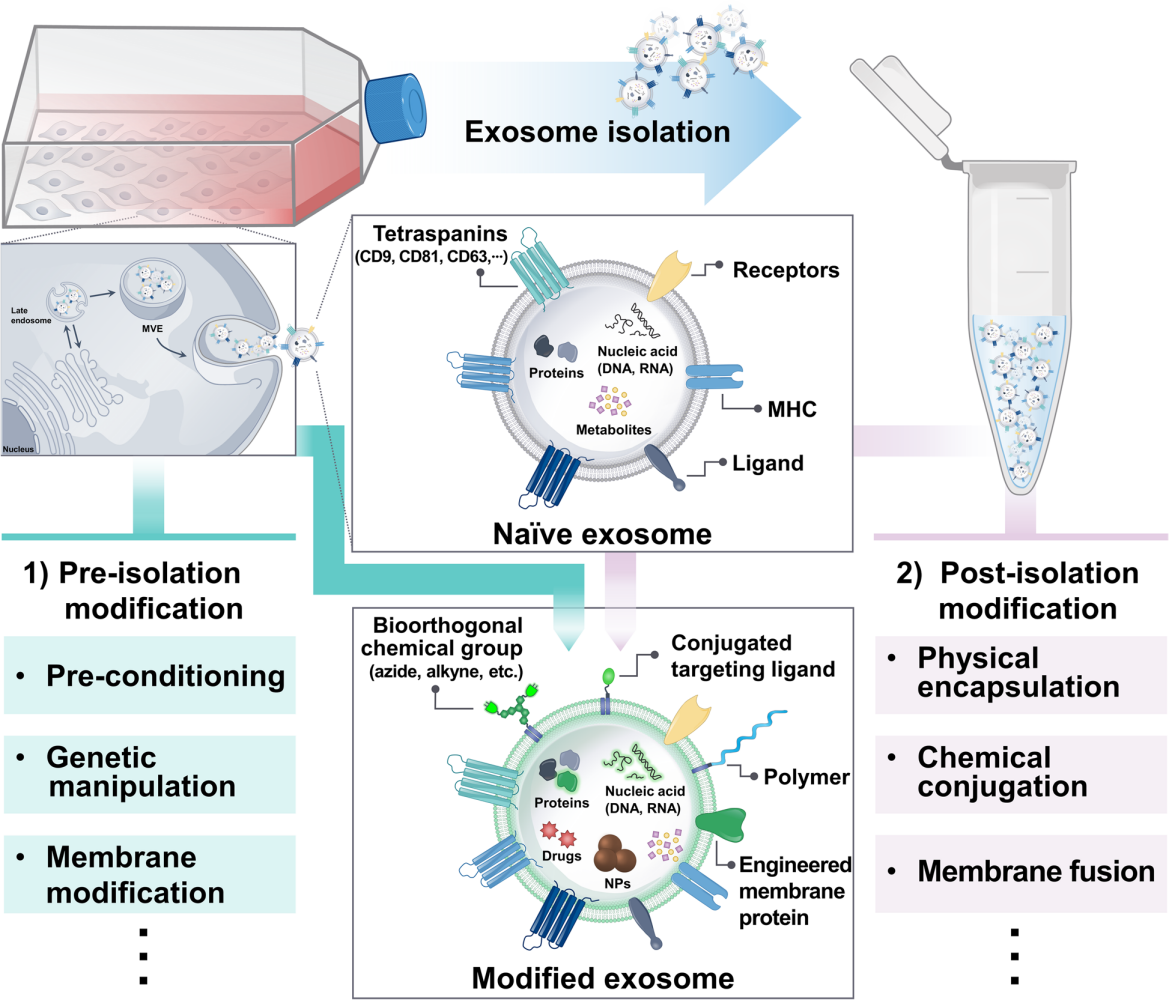
Exosome engineering via pre-isolation modification and post-isolation modification

Growth in the knowledge of the intrinsic biological functions of exosomes has led to the development of various exosome engineering strategies with biomedical applications. Considering these inherent biological functions of exosomes, bare-state exosomes from various types of cells were utilized to treat intractable diseases, including cancer, RA, multiple sclerosis, and diabetes [87–89]. Although exosomes have emerged as a replacement for cell-based therapy owing to their remarkable therapeutic efficacy, the regulation of exosomal compartments has been attempted to maximize their effects. Although exosomes possess homing effect, which can be recruited to parent cells, several engineering strategies have been proposed that disadvantages such as low targetability and poor distribution have still been issued. The engineering strategies can be classified into two categories: modification before and after the isolation of exosomes (Fig. 2).

#### Pre-isolation modification

**Fig. 3:**
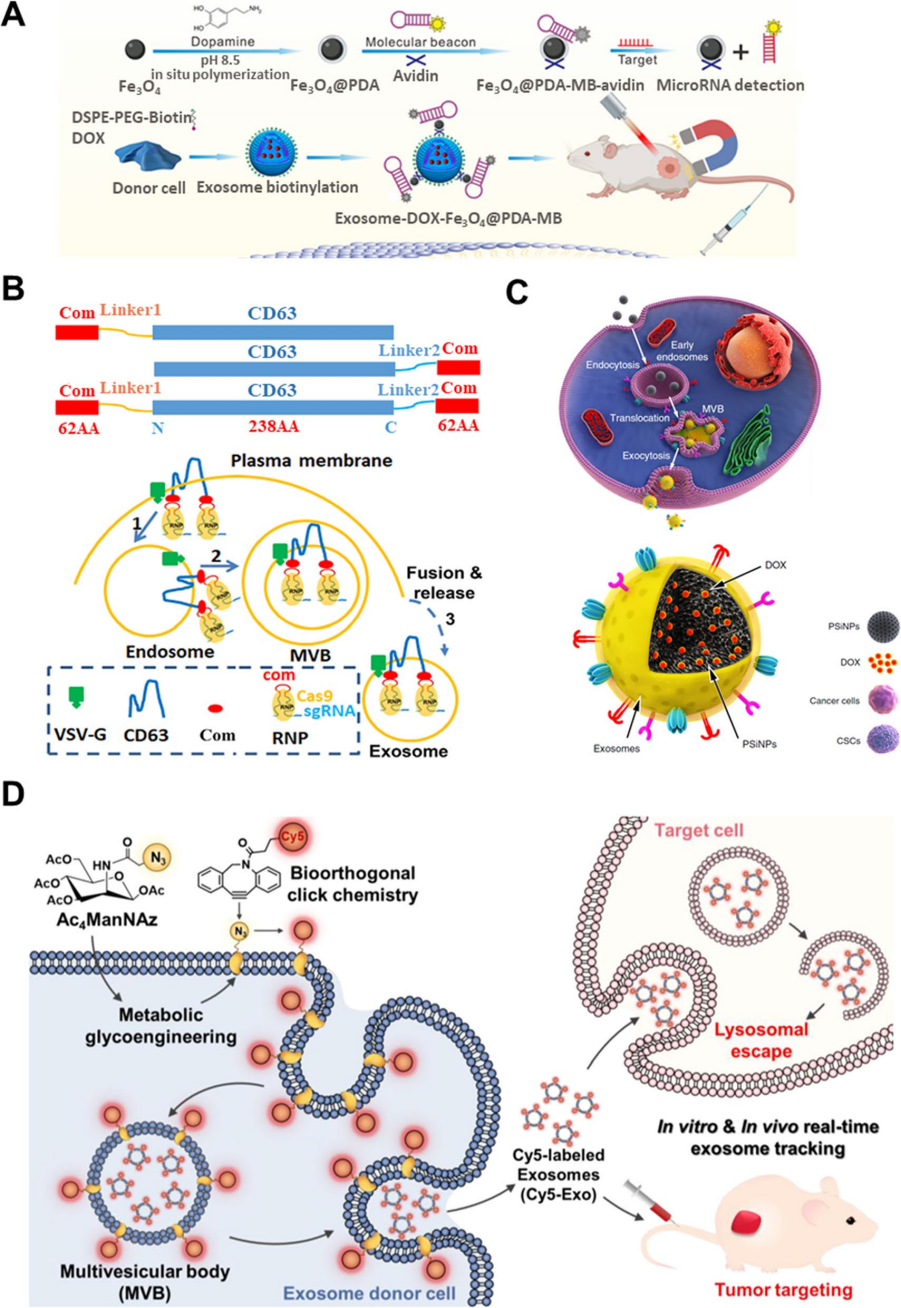
Schematic illustration of pre-isolation modification methods. **A** Exosomes from target ligand (avidin) labeled and anti-tumor drugs encapsulated donor cells for further labeling with avidin complex [90]. Reproduced with permission, Copyright 2021, Elsevier. **B** Fusion protein modulation in parent cells for enriching ribonucleoproteins (RNPs) in exosomes [91]. Reproduced with permission, Copyright 2021, Wiley. **C** Preparation of exosomal membrane-coated DOX@PSiNPs by exocytosis following DOX@PSiNPs incubation in parent cells [92]. Reproduced with permission, Copyright 2019, Springer Nature. **D** Dye-labeled exosomes from dye-labeled metabolic glycoengineered donor cells via biorthogonal click chemistry [93]. Copyright 2020, American Chemical Society

Exosomes can achieve the distinct properties of parent cells by loading the same protein, RNA, and DNA with origin cells as well as by expressing the membrane protein of secreted cells in the exosomal surface [1]. Accordingly, based on exosome biogenesis, the engineering methods for modulating parent cells are developed. The focus of the research includes pre-conditioning methods for altering cell characteristics using various cytokines or stimuli, endogenous modulation for regulating genetic parts, cell membrane modification for exosomal membrane surface engineering, and pre-treatment for cargo modification via the utilization of exosome biogenesis pathway (Fig. 3).

The innate characteristics of exosomes can be altered based on the status of parent cells, which are affected by external factors. Immune cells such as DCs and macrophages can differentiate into pro- or anti-inflammatory phenotypes via pre-conditioning during cultivation, depending on growth factors and cytokines. It is noted that the exosomes from those differentiated immune cells can alter the systemic immune system, which facilitates reversing the pro- or anti-inflammatory state. Pro-inflammatory mature DCs or anti-inflammatory tolerogenic DCs are induced by treating LPS/IFN-γ/tumor antigen or TGF-β/IL-10 to immature DCs, respectively. Although mature or tolerogenic DCs have been utilized as cancer vaccines or for autoimmune disease treatments, DC-derived exosomes (Dex) have replaced DCs for therapeutic use owing to the advantage that peptide-MHC II complexes are more Dex-rich than DCs. Owing to the intrinsic properties, mature Dex are used in developing cancer vaccines [94], as therapeutics for remyelination [95], and migraine [96]; however, tolerogenic Dex are used in the treatment of several autoimmune diseases, including multiple sclerosis [97], inflammatory bowel disease [98–100], and RA [80, 101–104].

Macrophages can differentiate into M1 or M2 via the pre-conditioning of pro-inflammatory cytokines like LPS/IFN-γ or anti-inflammatory cytokines such as IL-4/IL-13, respectively. M1-derived exosomes, which can reprogram tumor-associated macrophages into antitumorigenic M1-like macrophages, are used in cancer immunotherapy [105]. Moreover, it is used in developing cancer vaccines because M1-derived exosomes can form the pro-inflammatory niche in lymph nodes [106]. Unlike M1-derived exosomes, M2-derived exosomes facilitate the reprogramming of M1 to M2, reflecting anti-inflammatory properties. Owing to intrinsic properties similar to that of M2, M2-derived exosomes are used as therapeutics for various diseases, such as RA, wound healing [107, 108], and periodontitis [109], that require immunosuppressive effects.

Moreover, the immunomodulatory effects of the stem cells can be reinforced by pre-conditioning with growth factors and cytokines, and their exosomes have extensively been studied as therapeutics for various inflammatory diseases. LPS-preconditioned dental follicle stem cell-derived exosomes can ameliorate periodontitis via the ROS/MAPK signaling pathway [110]. TNF-α/IFN-γ-stimulated gingival mesenchymal stem cells (MSCs)-derived exosomes showed potent anti-inflammatory properties, representing exosome-mediated M2 polarization [111]. In addition, modifying the cultivation environment of stem cells can modulate the characteristics of stem cell-derived exosomes. Exosomes isolated from dental pulp and bone MSCs cultured in hypoxic conditions attenuated osteolysis by modulating macrophage polarization [112] and alleviated ulcerative colitis by preventing DNA damage and reactive oxygen species accumulation [113], respectively.

Exosome therapeutic efficacy can be partially attributed to their components. Strategies used for genetic manipulation, including transfection and transduction of cells, to maximize their therapeutic effects by upregulating the expression of relevant exosomal miRNA, DNA, RNA, and proteins have been considered as common engineering methods. Recently, tremendous interest in gene editing has led to the development of clustered regularly interspaced short palindromic repeats (CRISPR)/CRISPR associated protein 9 (Cas9)-engineered stem cells with high efficiency and precise genome editing in a targeted manner [114]. CRISPR/Cas9-engineered stem cells have been applied in clinical trials and have been also employed to obtain the genetically engineered exosomes. Wozowicz et al. scrutinized CRISPR/Cas9-engineered MSC-derived exosomes as carriers for designer nucleases (DNs) to delete target genes in recipient cells [115]. Although they also tried methods involving zinc finger nucleases (ZFNs) and transcription activator-like effector nucleases (TALENs), they focused on the CRISPR/Cas9 systems to obtain the engineered induced pluripotent stem cells (iPSCs)-derived exosomes which can knock-out the *Pcsk9* gene, overexpressed in hypercholesterolemia, in vivo and noted the ease of customizing the CRISPR/Cas9 system. To enrich the ribonucleoprotein and single guide RNA (sgRNA) into exosomes, the interaction between Com-CD63-Com and aptamer-binding protein-packaged Cas9-Com was introduced in HEK293T cells, and the effects of the isolated exosomes were examined in vitro and in vivo [91]. The most common strategy for developing exosomes using CRISPR/Cas9 carriers are loading methods for isolated exosomes.

In addition to the various methods for modulating the internal components of parent cells, the engineering strategies for exosomal membrane surface via modification of origin cells were reported. Owing to the presence of several proteins in cellular membranes, various components, such as targeting moieties and imaging agents for biomedical applications, can be conjugated through metabolic glycoengineering, bioorthogonal chemistry, and hydrophobic insertion.

Among the various types of bioorthogonal chemistry tools, click chemistry has been markedly utilized for molecular imaging in combination with metabolic glycoengineering, which can add the chemical groups on cellular glycans [116]. Theranostic nanoparticles and imaging probes such as fluorescent dyes were labeled onto the azide (N_3_) functional group via copper-free click chemistry utilizing metabolic glycoengineering using mannosamine. Although this strategy was mainly applied for in vivo tracking of several types of cells [117–119] and active-targeting of tumors [120], metabolic glycoengineering or bioorthogonal click chemistry was recently used for in situ one-step strategy to label exosomes, considering the exosome biogenesis pathway [93]. The authors noted that the yield of acquisition of dye-labeled exosomes from synthetic metabolite-treated donor cells was higher than that by applying bioorthogonal click chemistry directly to isolated exosomes, and also it preserved the intrinsic properties of exosomes.

In addition to the fluorescent dye conjugation, a study of exosomal surface-modified exosomes with biocompatible polymer via bioorthogonal chemistry based on metabolic glycoengineering was conducted. Park and group reported PEGylated hyaluronic acid (HA)-coated exosomes to target the CD44-overexpressing cells actively and verified the targetability using the RA and PC3 tumor-bearing mouse models, representative models of CD44-overexpression [121]. Briefly, the authors induced the expression of the N_3_ group in the cellular membrane of donor cells by pre-treatment with N-azidoacetyl-D-mannosamine (Ac_4_ManNAz), and the parent cells utilized in this research were cancer cells regarding the proof of concept. Subsequently, dibenzocyclooctyne-terminated PEGylated-HA was conjugated with the N_3_ group using bioorthogonal copper-free click chemistry. Therefore, the PEGylated-HA functionalized exosomes were isolated using ultracentrifugation, and surface editing was verified using biolayer interferometry (BLI) and confocal imaging. Thereafter, macrophage-targeing dextran sulfate (DS)-coated adipose-derived stem cells-derived exosomes for immunomodulation in RA were suggested by the same group [122]. The strategy for exosomal surface editing has been described previously; however, DS was introduced to target the macrophage scavenger receptor class A (SR-A) of activated macrophages.

Owing to the sophisticated physicochemical properties of phospholipid bilayer structures of cellular membranes, various functional moieties linked with lipid molecules can be incorporated into the cell membrane surfaces by hydrophobic insertion via physical interaction. Furthermore, this modification strategy can endow the exosomal membrane with functional moieties introduced into cells. Wang et al. amended the donor cells to obtain the dual ligand-modified and drug-encapsulated exosomes [123]. The authors pre-treated 1,2-distearoyl-sn-glycero-phosphoethanolamine (DSPE)-PEG-Biotin to introduce the biotin on cellular membrane surfaces, utilizing the physical interaction between DSPE and lipid bilayer of the cellular membrane. Subsequently, the cells were pre-treated with anti-tumor drugs, assuming that the drug will be packaged in exosomes following uptake into the cytosol. Although the anti-cancer drug paclitaxel (PTX) induced partial apoptosis, the authors noted that PTX was also well-encapsulated in exosomes. To edit the exosomes with dual ligands and enhance the targetability, Wang et al. subsequently treated donor cells with avidin, resulting in a biotin-avidin reaction, and successfully isolated these exosomes utilizing the microfluidic chips created by them. Wang et al. also applied the similar strategy to obtain biotinylated exosomes for further engineering with avidin modified with molecular beacon and poly dopamine (PDA) covered Fe_3_O_4_ nanoparticles [90]. Fe_3_O_4_ nanoparticles and molecular beacon decorated DOX-encapsulated exosomes simultaneously facilitated the magnet-targeted photothermal, chemotherapy, gene therapy, and molecular imaging. Even though the study reported by Chen et al. was about microparticles and not exosomes, the authors acquired the microparticles following the pre-treatment with DSPE-PEG-Biotin to harness the biotinylated microparticles for further modification [124].

The abovementioned strategies were associated with modifying the exosome itself, such as the internal contents or exosomal surface via pre-conditioning, whereas the studies relevant to drug delivery systems that utilize a part of the exosome are discussed here. Considering the homing effect of exosomes, various types of nanomedicines coated with exosomal surfaces were developed. While the commonly used methods for exosomal membrane coating onto the surface of nanoparticles were extrusion, electroporation, incubation, and sonication of isolated exosomes with nanoparticles, the pre-conditioning method has recently been proposed based on the exosome biogenesis pathway. Albero et al. compared several methods, including electroporation, passive loading, sonication, thermal shock, detergent-assisted loading, and pre-incubation to encapsulate hollow gold nanoparticles into melanoma cells-derived exosomes for photothermal therapeutics [125]. All methods, except pre-incubation, correspond to post-isolation modification, which is described in following section. Albero et al. noted that the yield of these strategies was only approximately 15%, whereas the yield of pre-incubation of the hollow gold nanoparticle was up to 50%. Pre-incubated nanoparticles are encapsulated within the multivesicular bodies (MVBs) following endocytosis. Because exosomes are produced by MVBs, endocytosed nanoparticles can be trapped in the newly produced exosomes by MVBs. Subsequently, the exosomal surface-modified nanoparticles can be obtained in the end. Hollow gold nanoparticle-loaded various types of cell-derived exosomes showed selective transfer only to the origin cells, reflecting the homing effects of exosomes [126]. Yang et al. developed tumor exosome-sheathed doxorubicin-loaded nanoparticles (DOX@E-PSiNPs) via exosome biogenesis pathway, and DOX@E-PSiNPs exhibited cytotoxicity in both cancer and cancer stem cells in vitro and in orthotopic and metastatic tumor models in vivo [92]. The strategy utilizing the exosome biogenesis pathway for nanoparticle surface modification can augment the yield without damaging exosomal surfaces, whereas the concentration of pre-treated nanoparticles has to be considered significant due to potential cytotoxicity.

#### Post-isolation modification

**Fig. 4:**
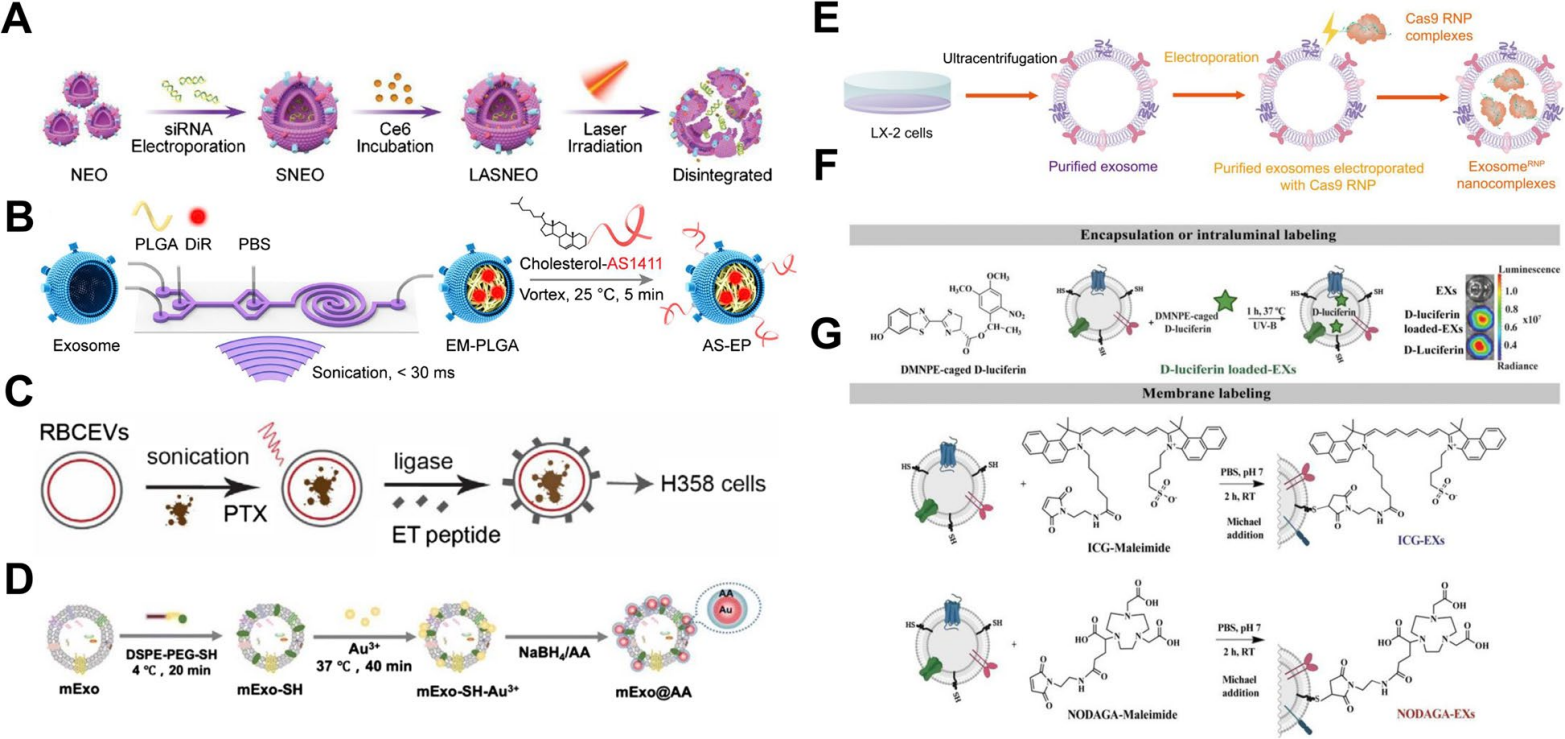
Schematic illustration of post-isolation modification methods. **A** Exosome loaded with siRNA using electroporation, then incubated with Chlorin e6 (Ce6) [129]. Copyright 2022, Wiley. **B** Exosome loaded with poly(lactic-co-glycolic acid) (PLGA) and DiR using sonication, then decorated with aptamer using vortex [130]. Copyright 2020, American Chemical Society. C Exosome loaded with paclitaxel (PTX) using sonication, then decorated with ET peptide [131]. Copyright 2021, Wiley. **D** Exosome decorated with DSPE-PEG-SH and Au^3+^ using incubation, then AA was introduced [132]. Copyright 2023, Royal Society of Chemistry. **E** Exosome electroporated with Cas9 ribonucleoprotein (RNP) [133]. Copyright 2022, American Association for the Advancement of Science. **F** exosome loaded with D-luciferin using incubation with UV-B. G exosome membrane labeled with ICG-maleimide and NODAGA-maleimide using Michael addition [134]. Copyright 2022, Wiley

Pre-isolation modification strategies for exosome engineering is challenging depending on the types of cells regarding the undesirable effects on parent cells with various treatments [127, 128]. In such cases, exosome engineering can be conducted by isolating exosomes. This review discusses the various strategies as post-isolation modification. The post-isolation modification methods were mainly utilized, 1) to utilize exosomes as carriers for various cargoes, such as chemical drugs, sonosensitizer, nanoparticles, and even proteins, 2) to facilitate prolonged blood circulation, 3) and to endow the functionalities like targetability and stimuli-sensitivity (Fig. 4).

As exosomes are nano-sized and contain lipid bilayer to encapsulate functional cargo, exosomes have been widely used as next-generation delivery vehicles for the past decade [127]. The feasible range of loading functional cargo can vary from small molecules to nanoparticles and proteins, making it flexible. Even though some of functional cargoes mentioned earlier can be encapsulated into exosomes via pre-isolation modification methods, but their unexpected degradation by either autophagy or destruction within lysosomes can be prevented through direct loading into isolated exosomes [135]. Moreover, by importing the cargo before the isolation of exosomes, unforeseen damages like apoptosis or changes in the original state of parent cells can be eliminated [136].

Small molecules such as chemical drugs; PTX, doxorubicin (DOX), erastin have been loaded in exosomes using sonication, incubation, extrusion, and dialysis methods for the treatment of various intractable diseases [137]. In addition to the conventional chemical drugs, small molecules such as an extract from natural compounds, photosensitizer, and sonosensitizer, have been loaded into isolated exosomes recently to use as therapeutic agents for chemotherapy (CDT), photodynamic therapy (PDT), sonodynamic therapy (SDT), and immunotherapy.

Cui et al. developed serum-derived exosomes loaded with natural products from Chinese medicine and surface-functionalized with CpG for glioma-targeted chemo-immunotherapy [138]. The extracts from Chinese medicine were tanshinone IIA (TanIIA) and glycyrrhizic acid (GL), which inhibits signal transducers and activates transcription 3 (STAT3); moreover, TanIIA and GL formed self-assembled nanomicelles. The exosomes encapsulated the nanomicelles using sonication methods in the ice bath for chemotherapy and were further modified with DSPE-PEG-CpG by incubation methods for immunotherapy. For the PDT combined immunotherapy, Zhang et al. reported Light-Activatable Silencing NK-Derived Exosome (LASNEO) by loading chlorin e6 (Ce6), and siRNA targeting PD-L1 into NK-derived exosomes [129]. Ce6 loaded via incubation and siRNA loaded via electroporation were released after laser irradiation, subsequently, inducing the reactive oxygen species (ROS) production and downregulation of PD-L1, respectively. Finally, the overproduced ROS, which can tempt the macrophage polarization into M1 type, and T cell activation by downregulated PD-L1 led to the immunotherapy post-laser irradiation. Several photosensitizers, which can act as sonosensitizer-loaded exosomes were demonstrated to facilitate the SDT. Cao et al. developed various SDT agents using HEK293T-derived exosomes. For active tumor-targeting SDT for breast cancer treatment, exosomes were surface-modified with folic acid and loaded with indocyanine green (ICG) via the incubation method [139]. Moreover, the authors encapsulated ICG, PTX, and sodium bicarbonate (SBC) into exosomes via incubation, and these facilitated the pH/ultrasound (US)-responsive chemotherapy and SDT [140]. Recently, with the same loading strategy, mitochondria-targeted SDT was conducted using HEK293T-derived exosomes loaded with mitochondria targeting moiety, triphenylphosphonium (TPP),-conjugated Ce6 and pro-oxidant chemodrug, and piperlongumine [141]. The authors used 4% (v/v) DMSO in PBS for loading and further noted a higher percentage of DMSO can lead to higher encapsulation yield by destroying the exosomes; this method might not be suitable for keeping the inner compartment, which can act as potential therapeutics, of exosomes.

To utilize the homing effects of exosomes according to the cell origin [65], exosomes from various cells have been widely used to modify the surface of theranostic nanoparticles via diverse strategies such as sonication, extrusion, and incubation. The hydroxychloroquine (HCQ)-loaded zinc sulfide (ZnS) nanoparticles, which act as a photosensitizer, were coated with glioblastoma cells-derived exosomes through thin film hydration followed by extrusion and subsequently modified with iRGD peptide which provided the pH- and redox- responsiveness [142]. This study noted that, owing to the homing ability of exosomes, HCQ@ZnS@eRGD was permeable across the blood–brain barrier and showed enhanced targetability to glioblastoma cells in vivo. In addition to the inorganic nanoparticles, exosome-coated poly(lactic-co-glycolic acid) (PLGA) nanoparticles loaded with 1,1-dioctadecyl-3,3,3,3-tetramethylindotricarbocyaine iodide (DiR) were obtained using microfluidic sonication and exhibited a prolonged blood circulation, suggesting the effects of exosomal surfaces [130].

Even though the endogenous modulation of origin cells facilitated the engineering of exosomal proteins and genes, they can be loaded into isolated exosomes by controlling the loading efficiency from the protein or gene delivery view. Wan and Zhong et al. reported a study related to tissue-specific gene therapy of liver fibrosis using Cas9 ribonucleoprotein (RNP) delivery exosomes [133]. The exosomes were isolated from LX-2 cells through ultracentrifugation, and Cas9 RNP complexes were loaded into exosomes via electroporation with 20% entrapment efficiency. Meanwhile, the human umbilical cord MSC-derived exosomes modified with polypeptide (CAQK peptide) and loaded with the CRISPR/Cas9 components were applied for the immunotherapy of spinal cord injury [143]. The CAQK peptide was chemically conjugated on exosomal surfaces via EDC/NHS chemistry, and CRISPR/Cas9 plasmid was loaded using electroporation methods. In addition to the electroporation strategy, McAndrews et al. used the commercially available transfection reagent, Exo-Fect Exosome Transfection Kit, to encapsulate the CRISPR/Cas9 plasmid DNA into MSC-derived exosomes for oncogenic Kras targeted therapy [144]. Lately, D. Hade et al. reported a novel microRNA loading strategy using cell-penetrating peptide [145]. The authors incubated the exosomes with the conjugation of miR-21-5p with a known cell-penetrating peptide, YARA, and noted that the loading efficiency improved 18.6-folds compared to only miR-21-5p incubation.

As the exosomes exhibit rapid clearance in vivo and most of them are accumulated in the liver, enhancement of prolonged blood circulation and targetability to lesions remain challenging [146]. Several strategies for surface engineering of exosomes have been investigated to solve these issues over the past few decades. Various modifications to the exosomal surface are available via either chemical reaction to form covalent bonds or physical interaction like lipid insertion, affinity-based interaction, enzymatic ligation, and fusion because exosomes possess numerous proteins and functional groups on exosomal surfaces and intrinsic lipid bilayer structures.

PEGylation is the most widely known prolonged blood circulation strategy, which takes advantage of PEG corona from interaction with biological molecules [147]. In addition to the PEGylation, several studies helped understand that CD47, an exosomal transmembrane protein which protects cells from phagocytosis by mononuclear phagocyte system, -upregulated exosomes to prolong blood circulation as a novel approach [148]. Cheng et al. designed exosome-liposome hybrid nanovesicles that exhibited prolonged blood circulation for photothermal therapy and immunotherapy [149]. The CD47-upregulated exosomes were isolated from plasmid-engineered CT26 cells, and fused with ICG and R837 encapsulated-thermosensitive liposome via the freeze-thaw method. The overexpression of CD47 on hybrid nanovesicles facilitated prolonged blood circulation by sending the “do not eat me” signal. Another hybrid nanocarrier composed of CD47-enriched human serum-derived exosomes and DOX and gefitinib (GE) co-loaded/RGD-expressed liposome for lung tumor-targeted therapy was demonstrated by Z. Belhadj et al. [150]. The hybrid nanocarriers were prepared by thin film hydration and extrusion method between exosomes and liposomes following exosome isolation from human serum through ultracentrifugation and manifested 120.96% lower endocytosis compared to only liposomes. Since the mononuclear phagocyte system (MPS)-mediated elimination of drugs has been addressed as an issue in the development of nanomedicines, this strategy to enhance the prolonged in vivo circulation time and tumor accumulation while attenuating the accumulation to the liver may act as a crucial point for approval in clinical practice.

In addition to modulating the biodistribution of exosomes, surface modifications with targeting, stimuli-sensitive, therapeutic, or imaging moieties to enhance the therapeutic efficacy or endow the imaging capability are discussed here.

Although exosomes exhibit the homing effect to origin cells, several studies have tried to add various kinds of targeting moieties to exosomes. For instance, the epidermal growth factor receptor (EGFR) was introduced on exosomal surfaces for reinforced targetability, considering that lung cancer is EGFR-positive. Pham et al. developed an enzymatic method for conjugating EGFR peptide onto exosomal surfaces [131]. The EGFR peptide was covalently bound to the red blood cells (RBC)-derived exosomes using Sortase A or OaAEP1 ligase, the protein ligating enzymes, at a neutral pH without causing any damage to the exosomes. In contrast, Huang et al. utilized the hydrophobic insertion method to incorporate EGFR aptamer onto kiwi fruit-derived exosomes [151]. Exosomes targeting EGFR-mutant non-small cell lung cancer were prepared by hydrophobic interaction between fluorophore/EGFR aptamer-linked cholesterol and lipid bilayer of exosomes, following the encapsulation of STAT3 siRNA.

To augment the targetability, the distinct properties of the disease-microenvironment, like acidic pH, high concentration of ROS and GSH, and overexpressed enzymes, can be used for developing stimuli-responsive nanoparticles for drug delivery [152]. Even though several stimuli-sensitive drug delivery systems, which facilitate active and passive targeting, have been evaluated, their clinical application remains to be validated in various experimental and preclinical systems. Lee and Sul evaluated reactive oxygen species-responsive tolerogenic exosomes for RA treatment since ROS is overproduced in the inflamed joints [153].

Ma et al. explored chemical reaction-based novel surface-modification strategy, which can be used in situ to modify the ascorbic acid (AA), an antioxidant, on the exosomal surface to overcome the low loading efficacy of common loading methods [132]. In this study, mesenchymal stem cell-derived exosomes were isolated using ultracentrifugation and incubated with DSPE-PEG-SH followed by incubation with HAuCl_4_ solution to introduce Au^3+^ on exosomal surfaces. Because AA can act as a reducing and protective agent for AuNPs growth, AA solutions were incubated with Au^3+^-introduced exosomes. Subsequently, exosomes with their surface-modified with AA-protected AuNPs (mExo@AA) were obtained and used as eye drop formulation to treat dry eye disease. Its therapeutic effects were achieved by eliciting damage repair, ROS scavenging, and macrophage polarization into the M2 type.

For gene/chemo/photothermal therapy and molecular imaging, a combination system composed of polydopamine (PDA) coated magnetic Fe_3_O_4_ nanoparticles and DOX-loaded exosomes was prepared using various exosome engineering strategies, including pre-isolation modification and affinity-based reaction [90]. Briefly, Fe_3_O_4_ nanoparticles were coated with dopamine by in situ polymerization to enhance the photothermal effect. Then, molecular beacon (MB) and avidin were fabricated on the surface of Fe_3_O_4_@PDA targeting miR-21 for molecular imaging and interaction with exosomes, respectively. Biotinylated and DOX-loaded exosomes were isolated from DSPE-PEG-Biotin and DOX-pretreated donor cells, and subsequently exosomes with their surfaces functionalized with Fe_3_O_4_@PDA-MB were generated by the reaction between the biotin of the exosome and avidin of Fe_3_O_4_@PDA-MB. Once the system entered the tumor cells, molecular imaging and silencing of miR-21 was facilitated by dequenching of MB, and chemotherapy/ photothermal therapy was enabled by DOX-released from exosome and Fe_3_O_4_ nanoparticles, respectively.

The exosome-based positron emission tomography (PET) imaging tool for diagnosing lung metastasis was developed by Almedia et al. [134]. The authors used osteosarcoma-derived exosomes for their homing ability, and conjugated the macrocyclic chelator NODAGA using the Michael addition between -SH functional group of exosomal surface and maleimide of NODAGA. Subsequently, the radionuclide ^64^Cu was labeled through a radiometal complexation. As the authors mentioned, even though the autologous exosomes from cancer patients bestow biocompatibility and non-immunogenicity and are not expected to induce protumoral effects, the safety issue should be considered indeed.

## Exosome-based therapeutic strategies for RA

The current treatment strategies for RA are based on the use of conventional drugs, including NSAIDs, DMARDs, and biological agents. Because the range and severity of RA symptoms are extensive and vary for each patient, DMARDs combination therapy is a potential alternative. In the past decades, biological agents that target cytokines such as TNF-α, IL-1, IL-6, T cells, and B cells, as well as signaling pathways such as Janus kinase (JAK) and mitogen-activated protein kinase (MAPK) have been identified. Recently, the treat-to-target strategy was reported to have achieved improved clinical outcomes [154]. However, some combinations of drugs are not recommended, and occurrence of adverse reactions, opportunistic infection, and two or more comorbidities remain challenges to overcome.

Considering the immune dysregulation in RA pathogenesis, cell-based therapy has emerged as a promising therapeutic approach. Even though the improved efficacy has been shown in several clinical trials, the limitations including storage, immunogenicity, and difficulty in controlling good manufacturing practices (GMPs), remain major obstacles. Over the past decades, innately therapeutic exosomes have attracted tremendous attention as a replacement for cell-based therapy. This section details the studies relevant to therapeutic applications of exosomes with or without engineering for RA treatment (Table 1).

**Table 1:** Therapeutic exosomes for treatment of RA

Origin	Target cell	Engineering method	Engineering purpose	Engineering modality	Animal model	Effect / Mechanism	Ref
Mouse bone marrow MSC	FLS	Bare	N/A	N/A	Wistar Rat RA model	TNF-α, IL-6, IL-8 ↓ FLSs proliferation ↓ Bax, PUMA, cytochrome C ↑ Negatively target cyclin I and activation of ATM/ATR/p53 signaling pathway Arthritis score ↓	[155]
Human bone marrow MSC	FLS	Bare	N/A	N/A	CIA	IL-1β, IL-6, IL-8 ↓ Negatively target CXCL9 and suppression of activation, migration, and invasion of FLSs	[156]
Human gingival MSC	T cell	Bare	N/A	N/A	CIA	CD4^+^IL-10^+^ T cells, IL-10 ↑ CD4^+^IFNγ^+^Th1, CD4^+^IL-17A^+^Th17, IFN-γ, IL-17A ↓ Incidence of arthritis and bone erosion ↓ IL-17RA-Act1-TRAF6-NF-κB signaling pathway ↓ Arthritis score ↓	[157]
Human umbilical cord MSC	T cell	Bare	N/A	N/A	N/A (RA patient)	Regulation of imbalance of Treg/Th17 IL-17 ↓ TGF-β ↑	[158]
Human umbilical cord MSC	T cell	Bare	N/A	N/A	CIA	RORγt ↓, Foxp3 ↑ Th17 cells, IL-17 ↓ Treg cells, IL-10, TGF-β ↑ Arthritis score ↓	[159]
Bovine milk	T cell	Bare	N/A	N/A	Polyarthritis (IL-1Ra^−/−^), CIA	TNF-α, MCP-1 ↓ Foxp3^+^ Tregs, TGF-β ↑ Delay the onset of IL-1Ra^−/−^ polyarthritis and CIA Regulation of Th1, Th2, Treg, and Th17 cells in both gut and spleen	[160]
Granulocytic-myeloid derived suppressor cell from CIA mice	T cell	Bare	N/A	N/A	CIA	Symptoms of RA ↓ Th1, Th17 cells, IFN-γ, IL-17A ↓ Th1↓via exosomal miR-29a-3p Th17 ↓ via exosomal miR-93-5p Arthritis score ↓	[161]
Mouse bone marrow MSC	T cell B cell	Bare	N/A	N/A	CIA, DTH	CD4^+^IL-10^+^ Tr1, CD4^+^CD25^+^Foxp3^+^ Treg ↑ CD8^+^IFNγ^+^ Cytotoxic T cells, CD4^+^IFNγ^+^ Th1 ↓ Plasmablast differentiation ↓ IL-10^+^ Breg ↑ Arthritis score ↓	[162]
Granulocytic-myeloid derived suppressor cell from tumor-bearing mice	B cell	Bare	N/A	N/A	CIA	Symptoms in CIA ↓, IgG and anti-CII antibody ↓ IL-10^+^ B cells ↑, plasma cells and follicular Th cells ↓ Phosphorylation of GSK-3β ↑ via exosomal PGE2 Arthritis score ↓	[163]
Human MSC	FLSs	Genetic manipulation	miRNA overexpression	miR-124a	N/A	MH7A, RA FLSs cell lines, proliferation ↓ Cell cycle arrest at G0/G1 phase Migration and invasion of MH7A ↓ Apoptosis of MH7A ↑ (Bax, Bid, Bim, Bcl-2, Caspase-3, Caspase-9 ↑)	[164]
Rat bone marrow MSC	FLS	Genetic manipulation	miRNA overexpression	miR-192-5p	CIA	RAC2 ↓ via exosomal miR-192-5p TNF-α, IL-1β, PGE2, NO, iNOS↓ Arthritis index ↓	[165]
Mouse bone marrow MSC	FLS	Genetic manipulation	miRNA overexpression	miR-150-5p	CIA	MMP14, VEGF↓via exosomal miR-150-5p Migration and angiogenesis of FLSs ↓ Arthritis index ↓	[166]
Human embryonic kidney 293 cell (HEK 293)	Macrophage	Genetic manipulation	Protein overexpression	IL-4	CIA	Localication of IL4Rα to early endosome ↑ M1 marker (TNF-α, CXCL10, iNOS, NO)↓ M2 marker (Arg, CD206) ↑ Arthritis index ↓	[167]
Bone marrow-derived DC	DC	Genetic manipulation	Protein overexpression	FasL	CIA, DTH	Internalize into CD11c^+^ DCs FasL-dependent effect MHC II-dependent effect Immunosuppressive in allogeneic mice Arthritis index ↓	[102]
Bone marrow-derived DC	DC, Macrophage, T cell	Genetic manipulation	Protein overexpression	IL-4	CIA, DTH	Internalize into CD11c^+^ DCs, F4/80^+^ macrophages Partially FasL-dependent effect MHC-dependent effect Effect on CD11c^+^ DCs, CD3^+^ T cells Arthritis index ↓	[103]
Bone marrow-derived DC	DC	Genetic manipulation	Protein overexpression	IDO, CTLA-4Ig	CIA, DTH	B7 molecules (CD80, CD86)-dependent effect IDO-mediated depleted tryptophan-dependent effect Arthritis index ↓	[104]
Bone marrow-derived DC	T cell	Genetic manipulation Preconditioning	DC differentiation	IL-10	CIA, DTH	T cell proliferation ↓ in vitro MHC II-dependent effect Immunosuppressive with intact morphology Arthritis index ↓	[80]
Macrophage	Macrophage	Preconditioning	Macrophage differentiation	IL-4	CIA	Favorable uptake into macrophages iNOS, TNF-α, IL-6 ↓, and Arg-1, CD163, TGF-β, IL-10 ↑ in reprogrammed M2 In situ reprogramming of M1 to M2 in synovial tissue Arthritis index, bone erosion ↓	[168]
Human bone marrow MSC	T cell	Preconditioning	Hypoxic, Normoxic, Pro-inflammatory	O_2_ portion, Pro-inflammatory cocktail	AIA	Arthritis index, joint swelling ↓ Synovial infiltrate ↓ (only in EV-NormO_2_) Th17 ↓ (only in EV-2%O_2_, EV-NormO_2_) Treg:Th17 ↑ (only verified in EV-NormO_2_)	[169]
Adipose-derived stem cell	Macrophage	Metabolic glycoengineering-mediated bioorthogonal click chemistry	Surface modification	Dextran sulfate	CIA	Cellular uptake into activated macrophages ↑ Accumulation into inflamed joints via SR-A binding ↑ Reprogramming M1 to M2 (iNOS ↓, CD206 ↑) Reprogramming M1 to M2 ↓ via miRNA inhibitor Arthritis index ↓, Synovial infiltration ↓ IFN-γ, TNF-α, IL-6, G-CSF, GM-CSF, IL-1β, IL-17A ↓ IL-4, IL-10 ↑	[122]
Human neutrophil	Arthritic cartilage, Chondrocyte	Sonication	Antibody incorporation	Anti-ROS-CII, Anti-ROS-CII-vIL-10, Anti-TNFα	AIA	Specific localization in arthritic joints Pro-inflammatory gene (*Tnf, Il-1β, IL-6, Mmp13, Adamts5*) ↓ Anti-inflammatory gene (*In-4, IL-10, Sox9, Acan, Col2a1*) ↑ Complete resolution of joint swelling	[170]
Macrophage	Chondrocyte	Sonication	Encapsulation	EGCG	CIA	HIF-1α ↓ Chondrocyte apoptosis ↓ (Cytochrome C, Caspase-3, Caspase-9, Bax, Bcl-2↓) Cartilage type II collagen ↑ Arthritis score ↓	[171]
Platelet	DC, Macrophage, T cell	Incubation	Encapsulation	Berberine	CIA	Macrophages, DCs activation ↓ Accumulation in inflamed joints CD45^+^ cells, T cells, macrophages, DCs ↓ Bone erosion, arthritic score ↓ Regulation of cytokines	[172]
Adipose human MSC	Synoviocyte	Incubation	Encapsulation	Curcumin	N/A	Acidic pH triggered faster Curcumin release Apoptosis of LPS-stimulated synovial cells ↑ IL-6, TNF-α, MMP-1, PGE2 ↓	[173]
Macrophage	Macrophage	Electroporation Hydrophobic insertion	Encapsulation, Surface modification	Dexamethasone, FA-PEG-Chol	CIA	Acidic pH triggered faster Dexamethasone release Cellular uptake into activated macrophage ↑ TNF-α and IL-1β ↓, IL-10 ↑ in vitro and in vivo Accumulation in the joints ↑ Bone erosion ↓ No hepatotoxicity	[174]
DCs	DC, T cell	Preconditioning, Hydrophobic insertion	DC differentiation, Surface modification	IL-10, DSPE-TK-mPEG	CIA	Selective cellular uptake into mature DCs via cleavage of ROS-sensitive TK linker on exosomal surface CD40, TNF-α, IL-6, CD4^+^ T cells ↓ Prolonged blood circulation, accumulation in joint ↑ IL-16 ↓, TGF-β and Treg ↑	[153]
Neutrophil	FLS, Chondrocyte Macrophage	Preconditioning, Bioorthogonal click chemistry	Protein upregulation, uPB incorporation on exosomal surface	LPS, uPB	CIA	Cellular uptake into activated FLS, chondrocyte, and macrophage ↑ Apoptosis of activated FLS, chondrocyte, and macrophage ↓ Protection against oxidative stress via SOD-2 mimics Accumulation into inflamed joints↑ Treg ↑ and Th1 ↓ Arthritis index, bone erosion ↓	[175]
Macrophage	Macrophage	Preconditioning, Transfection, Electroporation	Macrophage differentiation, Protein expression, Encapsulation	IL-4, IL-10 pDNA, BSP	CIA	Macrophage polarization from M1 to M2 Enhanced cellular uptake into M1 iNOS, IL-12 ↓ and CD206, Arg-1 ↑ Accumulation into arthritic joints ↑ Arthritis index ↓ Serum IL-1β, TNF-α ↓ and IL-10, IL-4 ↑ No hepatotoxicity	[176]
Macrophage	Macrophage	Electroporation, Exogenous stimuli	Encapsulation, Enhancement of targeting effect	IL-10, US	CIA	Cellular uptake into LPS-activated macrophage upon US irradiation ↑ Arthritis index, bone erosion ↓ IL-6, TNF-α ↓ CD86/CD206 ratio↓(CD206↑)	[177]
Macrophage	Macrophage	Chemical binding, Ultrasonication	Enhanced cellular uptake, Encapsulation	Polyarginine peptide, Curcumin	CIA, SCI	Cellular uptake into M1 macrophage ↑ Accumulation into inflamed joints ↑ CD206 ↑ Bone erosion, arthritic score ↓	[178]

*AIA* Antigen-induced arthritis, *BSP* Betamethasone sodium phosphate, *Chol* Cholesterol, *CIA* collagen-induced arthritis, *CTLA4-Ig* Cytotoxic T lymphocyte-associated antigen-4-Ig, *CXCL9* C-X-C motif chemokine ligand 9, *DCs* dendritic cells, *DSPE* 1,2-distearoyl-sn-glycero-3-phosphorylethanolamine, *DTH* Delayed hypersensitivity, *EGCG* Epigallocatechin gallate, *EVs* Extracellular vesicles, *FA* Folic acid, *FLSs* Fibroblast-like synoviocytes, *IDO* Indoleamine-2,3-dioxygenase, *iNOS* Nitric oxide synthase, *LPS* Lipopolysaccharide, *MSCs* mesenchymal stem cells, *NO* Nitric oxide, *PEG* Poly(ethylene glycol), *PGE2* Prostaglandin E2, *ROS* Reactive oxygen species, *TK* Thioketal, *uPB* Ultrasmall Prussian blue nanoparticles, *US* Ultrasound, *VEGF* Vascular endothelial growth factor, *SCI* Spinal cord injury

### Bare exosome

Rheumatoid arthritis microenvironment (RAM) is composed of various stromal cells, including fibroblast-like FLSs, T cells, B cells, endothelial cells (ECs), macrophages, and DCs. Without manipulation of parent cells or exosomes, bare exosomes derived from various sources can target specific cells in RAM and show promising efficacy in alleviating RA symptoms via microRNA transport, inducing signal transduction, and regulating immune responses.

FLSs are the most common non-immune cells in synovial tissue and they play a crucial role RA pathogenesis [179]. In addition, FLSs tend to proliferate in inflamed RA synovium, and their abnormal proliferation, migration, and invasion lead to RA progression [180]. Several microRNAs are downregulated in synovial tissue of inflamed arthritis and are packaged in exosomes derived from various types of stem cells. These findings support anti-inflammatory role of miRNA-contained exosomes. Several studies have reported the effect of specific miRNA-contained exosomes on FLSs in RA and their functions by predicting the target gene using TargetScan program. Wu et al. demonstrated the functions of miRNA-34a (miR-34a) contained mouse bone marrow stem cells (BM-MSCs)-derived exosomes in a rat model of RA [155]. Exosomes from BM-MSCs cultures (passage 3) were isolated using differential centrifugation methods and their therapeutic efficacy was explored in a rat model of RA. Evs localized in synovial tissues, and the anti-inflammatory effects were demonstrated using GW4869, an exosome biogenesis inhibitor. In particular, Evs inhibited FLS proliferation in the rat model of RA and increased miR-34a expression in FLSs. To study the role of exosomal miR-34a, the authors performed the miRNA gain- and loss-of-function experiments by transfecting BM-MSCs with miR-34a silencing or overexpressing systems. Presence of miR-34a in exosomes suppressed FLS proliferation as well as the secretion of pro-inflammatory cytokines in vivo. Moreover, the miR-34a-inhibited exosomes reduced the release of cytochrome C and downregulated the expression of caspase 3, Bax, PUMA, and cleaved poly (ADP-ribose) polymerase in FLSs, suggesting that exosomal miR-34a induced apoptosis. Furthermore, the mechanism involved in miR-34a function and downstream pathways were identified utilizing the web-based microRNA target detection program, Targetscan. The target gene was predicted, and a dual-luciferase reporter gene assay was performed. The data revealed that Evs negatively targeted cyclin I, an anti-apoptosis mediator, and subsequently activated ATM/ATR/p53 signaling pathway, resulting in FLS apoptosis and alleviation of RA inflammation. In another study, Meng et al. investigated the regulatory functions of exosomal miR-320a in FLSs obtained from RA patients (RA-FLSs) [156]. The authors selected the therapeutic target utilizing the Gene Expression Omnibus (GEO) database, EVmiRNA database, and TargetScan database. GEO database of normal versus RA-relevant disease samples allowed focus on the chemokine ligand 9 (CXCL9), which is overexpressed in RA. The miR-320a negatively targeted CXCL9 in RA-FLSs, thereby suppressing RA-FLSs activation, migration, and invasion. Furthermore, to identify the effects of BM-MSCs-derived exosomal miR-320a, the authors transfected miR-320a in BM-MSCs. Exosomes derived from miR-NC (control) or miR-320a transfected BM-MSCs suppressed CXCL9 expression, inhibited the activation and migration of FLSs, repressed inflammatory cytokines in vitro*,* and alleviated RA symptoms. Despite the data showed significant statistical differences, the exosomes derived from BM-MSCs without transfection showed a similar tendency to those with transfection. Therefore, the effect of upregulated exosomal miR-320a has been emphasized. Even though Wang et al. did not examine the therapeutic effect of exosomes, the impact of platelet-derived exosomes (PEVs) in FLSs has been reported [181]. The PEVs enhanced the migration and invasion of FLSs via a CXCR2-mediated activation of the NF-κB signaling pathway. Therefore, PEVs relevant to CXCL7/CXCR2 may be promising therapeutic targets for RA. In contrast, Chen et al. demonstrated that RA FLSs-derived exosomes promoted angiogenesis in ECs [182]. Exosomal miR-1972 was transferred to the ECs, which led to the downregulation of p53 and regulation of phosphorylated mTOR. Since blocking miR-1972 abrogated angiogenesis, which in turn can inhibit RA progress, the authors highlighted targeting RA FLSs-, LPS-activated FLSs-derived exosomes, or miR-1972 for treating RA. In the view that abnormal proliferation and augmented invasion of FLSs in RAM exacerbate the symptoms of RA by producing more pro-inflammatory cytokines, interacting with immune cells, and forming angiogenesis, switching the behavior of dysregulated FLSs with exosomes might be promising strategies.

T cells can recognize autoantigens during the initiation of RA pathogenesis. They play a pivotal role in the infiltration of various immune cells in inflamed joints of RA [183]. Not limited to the joint, T-helper (Th) 1 cell and Th17 cells are important effector T cell subsets involved in RA progression, whereas Treg cells acts as suppressors of RA [184]. Various types of cells and biological fluid-derived exosomes modulate T cell biology in RA, enhancing their therapeutic efficacy. Tian et al. demonstrated the immunosuppressive effects and mechanism of human gingival mesenchymal stem cells (GMSCs)-derived exosomes (GMSC-Exo) in CIA as latter regulate the T cell subsets [157]. The authors reported robust immunoregulatory capability of GMSC-Exo compared to other MSCs-exosomes; the GMSCs are exposed to a microenvironment enriched with diverse inflammatory factors. The GMSCs were obtained from healthy periodontium using differential centrifugation. GMSC-Exo suppressed the differentiation of CD4^+^ cells into Th1/Th17, and instead promoted their differentiation into Treg cells in vitro. Similarly, the in vivo data using a CIA model showed that GMSC-Exo induced the polarization of Th cells both in the spleen and lymph nodes, thereby alleviating inflammatory responses and inhibiting bone erosion. The elevated levels of IL-10 and suppressed levels of IFN-γ and IL-17A play key roles in therapeutic effect in RA considering that these cytokines can further activate or inhibit various types of immune cells such as B cells, Th2 cells, and neutrophils. Moreover, the authors verified that the immunosuppressive effect of GMSC-Exo was mediated via inhibition of the IL-17RA-Act1-TRAF6-NK-κB signaling pathway. Considering that the arthritic score was not significant at the time point of treatment despite final therapeutic efficacy, further evaluation of GMSC-Exo is necessary before their use in clinical settings. Even though Tian et al. did not mention the effects of GMSC-Exo on the macrophages, the polarization of the M1 phenotype to the M2 phenotype mediated via GMSC-Exo was reported to enhance the therapeutic efficacy in CIA [185, 186]. Zhang et al. evaluated human umbilical cord mesenchymal stem cells (hUCMSCs)-derived exosomes in autologous and allogenic models of RA [158, 159]. hUCMSCs offer tremendous advantages, including strong proliferation ability, varied sources, and low immunogenicity. The authors demonstrated the autologous immunoregulatory effect of hUCMSCs-exosomes (EVs) in RA patients compared to hUCMSCs [158]. Using EVs as a new treatment strategy was proposed after verifying their regulation of Treg/Th17 imbalance and pro- and anti-inflammatory cytokine secretion. In another study, these authors evaluated the immunomodulatory effects of hUCMSCs-exosomes (sEVs) in a rat model of allogeneic CIA [159]. sEVs alleviated the symptoms of RA by regulating pro- and anti-inflammatory cytokine secretion as well as T cell proliferation. Enhanced differentiation of T cells to Treg and suppression of Th17 at the transcription level were verified by downregulated RORγt level and elevated Foxp3 level. Based on these findings, the authors proposed sEVs as promising and safe substitutes for allogeneic MSCs, which can elicit anti-donor immunity. Arntz et al. showed the effects of bovine milk (BM)-derived exosomes (BMEVs) on spontaneous polyarthritis and a CIA model [160]. BM can improve inflammatory markers due to presence of several proteins with anti-inflammatory properties [187]. Moreover, the applications of BMEVs for treating inflammatory diseases were reported recently [188, 189]. Arntz et al. isolated exosomes from semi-skimmed pasteurized cow milk; BMEVs decreased TNF-α and MCP-1 levels, and increased the expression of Foxp3^+^ Tregsand TGF-β in vitro. Orally administered BMEVs in a mouse model of IL-1Ra-/- polyarthritis enhanced adaptive immunity in the gut by increasing the proportion of T-bet, IL-17, and CD4^+^Foxp3^+^ T cells, suggesting the BMEVs might regulate T cell plasticity via modulating gut microbiome. Furthermore, BMEVs in drinking water decreased the IgG and IgG2a (marker of Th1) while not changing IgG1 (marker of Th1), indicating that BMEVs also modulate the Th1/Th2 balance, which is critical in RA pathogenesis [190]. The suppressive effect of BMEVs on lipopolysaccharide (LPS)-activated macrophages exerted via NF-κB pathway might have positively influenced the therapeutic efficacy in RA [191]. Exosomes of other origins that exhibit T cell modulatory effect have also been proposed. Wang et al. evaluated the effects of granulocytic myeloid-derived suppressor cells (G-MDSCs)-derived exosomes (G-exo) in CIA [161]. MDSCs, which can be classified into granulocytic and monocytic populations, are a heterogeneous subset of myeloid cells, which greatly expand under various pathological conditions and possess robust immunosuppressive ability [192]. In this study, the authors generated CIA mice to isolate the G-MDSCs and monocytic GMSCs (M-MDSCs); the exosomes were isolated using ExoQuick-TC™. Similar to in vitro results, the G-exo alleviated RA symptoms by decreasing the proportion of Th1 and Th17 cells, while M-MDSCs-exosomes showed no considerable effects. For mechanistic evaluation, microRNA sequencing was performed, and the target gene was predicted utilizing TargetScan. The data revealed that miR-29a-3p and miR-93-5p contributed to the suppression of Th1 cells by targeting T-bet and Th17 cells by targeting STAT3, respectively.

T and B cells are implicated in the pathogenesis and progression of RA as they modulate the humoral response [193, 194]. Cosenza et al. compared the immunosuppressive effects of MSCs-exosomes (Exos) and MSCs-derived larger microparticles (MPs) on T and B cells [162]. Both Exos and MPs elevated the count of T regulatory type 1 (Tr1) cells and Tregs, and suppressed cytotoxic T cells and Th1 cells. In addition, both Exos and MPs inhibited plasmablast differentiation via modulating TGF-β1, PGE2, and IL1RA. Contrary to the results obtained in vitro*,* the counts of Th1, Th17, and Treg did not change, whereas those of Tr1 decreased, suggesting that in vitro experimental conditions may not always reflect the in vivo microenvironment. In CIA, Exos decreased the proportion of CD138^+^ plasmablasts and elevated CD19^+^IL10^+^ Breg-like population. Furthermore, the IL-1β was suppressed only in concanavalin A (ConA) activated lymph nodes, and IL-10 was augmented only in type II collagen-activated lymph nodes, indicating the antigen-specific anti-inflammatory effects. The decreased IgG2a/IgG1 ratio in Exos treated group revealed increased involvement of Th2 type humoral immune response, characterized by enhanced B cell proliferation and antibody production, and reduced Th1 type cellular immune response [195, 196]. Given that no significant differences in T cells were observed, the therapeutic efficacy of RA is possibly attributed to IL-10^+^ Breg cells, implying the importance of B cells in RA treatment. Wang et al. also studied the effects of G-exo on the humoral immunity in CIA [163]. They isolated the G-MDSCs from tumor-bearing mice and showed that different sources of G-MDSCs exhibit differential immune responses. The G-exo attenuated the symptoms in CIA and increased the proportion of IL-10^+^ B cells in both spleen and draining lymph nodes (dLNs), but decreased the proportion of the plasma cells (B220^−^CD138^+^) in dLNs and follicular Th cells (CD4^+^CXCR5^+^PD-1^+^) in both the organs. Considering that the plasma cells accumulate in RA and follicular Th cells are responsible for B cell maturation, these data suggest changes in those cells can contribute to the alleviation of RA symptoms [197–199]. Furthermore, decreased levels of IgG reflects altered status of humoral immunity. The mechanistic studies related to the production of IL-10-producing B cells were performed using a COX-2 inhibitor, and the results suggested that prostaglandin E2 (PGE2)-mediated GSK-3β signaling pathway is related.

Exosomes can influence the adaptive immune system and the innate immune system, including macrophages and DCs of RAM. You et al. demonstrated that MSCs-derived exosomes can reprogram the M1 macrophages to M2 macrophages [122]. Lee et al. verified that DCs-derived exosomes can downregulate the count of mature DCs and suppress inflammatory factors in CIA model [153]. Because those studies focused on the exosome engineering methods and highlighted the effects of engineered exosomes, they are discussed in following sections.

Microvesicles (MV), a subtype of extracellular vesicles, can either prevent inflammation in RA patients by modulating macrophages and FLSs or protect the cartilage by regulating chondrocytes. Perretti et al. investigated the activity of neutrophil-derived MV on human monocyte-derived macrophages and assessed the downstream crosstalk between macrophages and FLSs [200]. MV isolated from either TNF-α stimulated healthy human neutrophil or RA patients attenuated the classical activation of macrophages via the phosphatidylserine/MerTK axis and produced TGF-β via annexin A1 (AnxA1)/FPR2 axis. Subsequently, polarized macrophages suppressed the production of pro-inflammatory factors by FLSs. In another study, Perretti et al. reported that MV, which was enriched in AnxA1 and isolated from RA synovial fluid neutrophils also protected the cartilage degradation by producing TGF-β via FPR2/ALX signaling [201].

### Engineered exosome

Non-engineered exosomes are promising and safe substitutes for cell-based therapy. Nevertheless, the issues of poor targetability and long-term stability in the bloodstream remain to be overcome. Several studies have modified the exosomal membrane surface or their payload components such as miRNA, mRNA, and exosomal protein for enhancing their applicability. In this section, we have classified the engineered exosomes according to engineering methods discussed in previous section and further applications in RA are discussed.

#### Pre-isolation modification

The intrinsic properties of exosomes mirror the state of originated parent cells. Even though in some instances the exosomes possess immunomodulatory effects without any modulation of parent cells, use of diverse engineering methods on parent cells, including genetic manipulation, preconditioning, and metabolic engineering, can lead to the generation of exosomes with maximized therapeutic efficacy.

Exosomes derived from transfected or transduced parent cells can overexpress miRNA or protein, leading to their effective delivery to target cells and enhanced therapeutic efficacy. Meng et al. used human MSCs-derived miR-124a overexpressed exosomes [164], and the authors noted the necessity of carriers for therapeutic miRNA delivery to target cells. MSCs were selected as parent cells for their immunosuppressive effects, and miR-124a, known to regulate the FLSs in RA, was chosen as a target. Adenovirus transduction induced the overexpression of miR-124a in MSCs-exosomes as well as MSCs. Those exosomes inhibited the proliferation of MH7A, RA FLSs cell lines, and promoted apoptosis by arresting the cell cycle at G0/G1 phase. Zheng et al. transfected BM-MSCs to overexpress miR-192-5p in exosomes [165] and investigated its functions of suppressing FLSs in RA, using exosomes as miRNA carriers. miR-192-5p was successfully upregulated in BM-MSCs-exosomes following transfection with recombinant lentivirus particles and RAC2 was predicted as the target gene of miR-192-5p utilizing the TargetScan. The effects of upregulated exosomal miR-192-5p on either downregulation of RAC2 or therapeutic efficacy in rat models of CIA were compared to control miR-transfected BM-MSCs-derived exosomes; the results revealed that miR-192-5p alleviates RA pathological features. Chen et al. investigated the therapeutic potential of MSCs-derived miR-150-5p-overexpressing exosomes in RA [166]. Briefly, miR-150-5p, downregulated in FLSs of RA, was chosen as a therapeutic agent and loaded into MSCs-exosomes (known as anti-inflammatory mediators). The authors emphasized the regulation of expression of MMP14 and vascular endothelial growth factor (VEGF) on cartilage damage; further mechanistic studies were performed to elucidate the relation between miR-150-5p and MMP14 and VEGF in FLSs. The data showed that exosomal miR-150-5p suppressed MMP14 and VEGF in FLSs in vitro and in vivo, supporting the role of exosomes as effective delivery vehicles. Takenaka et al. developed the IL-4-carrying exosomes (IL-4-sEVs) for the effective delivery of IL-4 to macrophages for treating RA via macrophage polarization [167]. HEK293 cells were chosen as the parent cells of exosomes and transfected with plasmid DNA encoding lactadherin, exosome-tropic protein, and IL-4. The uptake of IL-4-sEVs was facilitated by recognizing negatively charged ligands on the exosomal surface by MSR1 of macrophages. The IL-4-sEVs enhanced IL-4 signaling in macrophages by promoting the localization of the IL4 receptor (IL4R) to the early endosome (EE). IL-4-sEVs-mediated polarization of M1 to M2 alleviated the symptoms in CIA. Robbin et al. modified BM-derived DCs (BMDCs) by adenoviral infection to express Fas ligand (FasL) [102], IL-4 [103], and indoleamine 2,3-dioxygenase (IDO) or cytotoxic T lymphocyte-associated antigen-4-Ig (CTLA-4Ig) [104] to differentiate immature DCs into tolerogenic DCs, which exert immunosuppressive effects. DCs transfected with adenovirus-encoded FasL as well as isolated exosomes possessed FasL, MHC, CD11c, and costimulatory molecules [102]. Exo/FasL was taken up by CD11c^+^ DCs, and the immunosuppressive effects depended on MHC class II and the Fas interaction. In CIA, intravenously injected Exo/FasL decreased the arthritis index. Furthermore, adenovirus-encoded secreted IL-4 (sIL-4) and membrane-bound IL-4 (mbIL-4) were transfected in BMDCs, and the immunosuppressive effects of DCs-derived exosomes (Exo/sIL-4, Exo/mbIL-4) was verified [103]. Both exosomes showed therapeutic effect in CIA, suggesting that the immunosuppressive effects were not solely mediated by sIL-4. Mechanism studies using the delayed-type hypersensitivity (DTH) model suggested that Exo/IL-4 were internalized into DCs, rendering them immunosuppressive and capable of directly or indirectly modifying T cells. Similarly, BMDCs were transduced with adenovirus-encoded IDO or CTLA-4Ig, an IDO inducer, and isolated exosomes exhibited immunosuppressive ability in CIA and DTH via IDO-mediated depletion of tryptophan in DCs and T cells as well as via B7 molecules-dependent pathway [104].

Priming the parent cells with biochemical stimuli can enhance immunomodulatory effects of cells as well as their derived exosomes. Kim et al. generated immunoregulatory BMDCs by adenoviral infection and preconditioning [80]. Adenovirus-encoded IL-10 (Ad.vIL-10) was used for genetic modulation, and the recombinant murine IL-10 (rmIL-10) was used to treat a culture of BMDCs before exosome isolation. Both exosomes exhibited immunosuppressive effects. Moreover, the data indicated that immunoregulatory effects of Exo/Ad.vIL-10 and Exo/IL-10 in CIA were due to the suppression of T cell proliferation and MHC class II-dependent pathway.

**Fig. 5:**
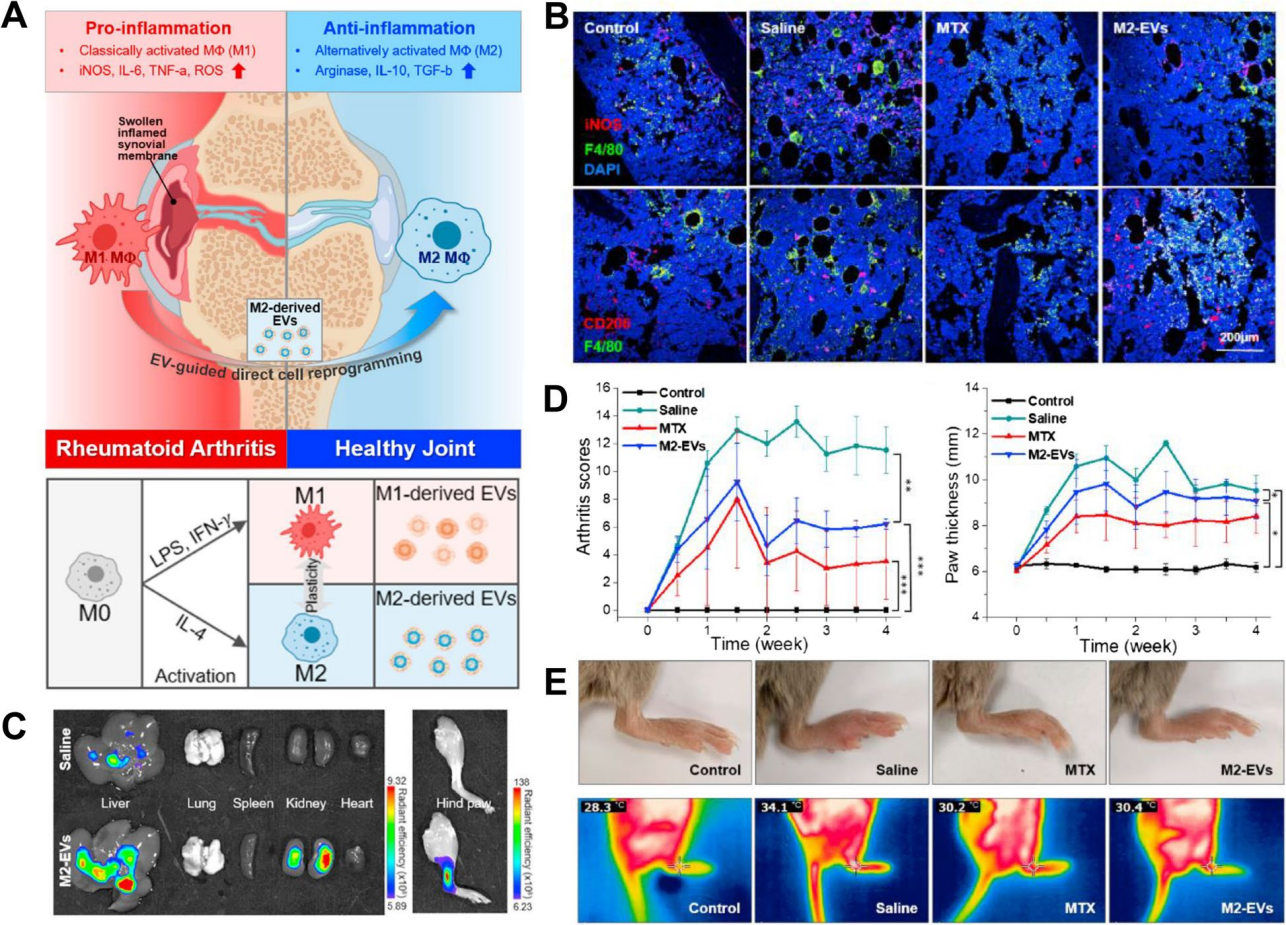
**A** Schematic illustration of RA treatment using M2 macrophage-derived exosomes, M2-EVs, (up) and preparation strategy (down). **B** Immunofluorescence images of inflamed joint post-injection of saline, MTX, and M2-EVs. **C** Ex vivo organ distribution images after footpad injection of saline and M2-EVs. **D** Clinical score and paw thickness of collagen-induced arthritis (CIA) mice after treatment of the samples. **E** Conventional and thermal images of hind paws in various treatment groups [168]. Reproduced with permission, Copyright 2022, Elsevier

Kim et al. developed M2 macrophage-derived exosomes (M2-EV) by preconditioning the culture medium of BM-derived macrophages (BMDM) to induce macrophage polarization (Fig. 5) [168].

To induce M2 macrophages, 20 ng/mL IL-4 was added as a media supplement for treating naïve M0 macrophages. The M2-EV facilitated the in situ macrophage polarization and re-established M1-M2 macrophage equilibrium; the conversion rate of M1 to M2 via M2-EV was over 90%, and reprogrammed M2 (RM2), possessed M2-like biological functions, as well as M2-specific proteins. Proteomic study verified that the major components in the reprogramming were MMP10, CCL8, glutamine synthetase, and MFG-E8. In CIA, the arthritis score of the footpad injected M2-EV was slightly higher than that injected with methotrexate (MTX); however, only M2-EV-treated group did not exhibit any damage in the intestine or hepatotoxicity. Moreover, M2-EV reprogrammed synovial resident M1 macrophages to M2 phenotype in situ, whereas MTX deprives both M1 and M2 macrophage in synovial tissue, indicating that long-term therapy with MTX may not be feasible. Kay et al. examined whether the exosomes from MSCs cultured in hypoxic, normoxic, and pro-inflammatory conditions showed therapeutic effects [169]. Hypoxic and normoxic conditions were generated by maintaining 2% and 21% oxygen, respectively, during cell culture. The pro-inflammatory condition was simulated by the addition of 10 ng/mL IFN-γ, 10 ng/mL TNF-α, and 5 ng/mL IL-1β. All exosomes reduced arthritis index and joint swelling, while only normoxic exosomes (EV-NormO_2_) suppressed synovial infiltration of immune cells. In addition, hypoxic exosomes (EV-2%O_2_) EV-NormO_2_ decreased the proportion of Th17 cells; however, this was not observed with pro-inflammatory conditioned exosomes (EV-Pro-Inflam) in vitro. Although histological changes did not improve in the antigen-induced arthritis (AIA) model when EV-2%O_2_ and EV-Pro-Inflam were used, Treg:Th17 cell ratio was restored with only EV-NormO_2_. Augmented Treg:Th17 ratio in vivo showed that the immunomodulatory effect might not depend on the priming methodology, although primed exosomes may have therapeutic potential. The downregulation of the co-stimulatory molecule and activation marker in macrophages was attributed to the therapeutic potential of EV-2%O_2_ [202]. Donate et al. showed that cigarette smoke induced miR-132 upregulation in Th17 cells-derived exosomes, and promoted osteoclastogenesis [203]. The authors noted that exogenous factors could influence immune cell-derived exosomes, aggravating RA progression.

**Fig. 6:**
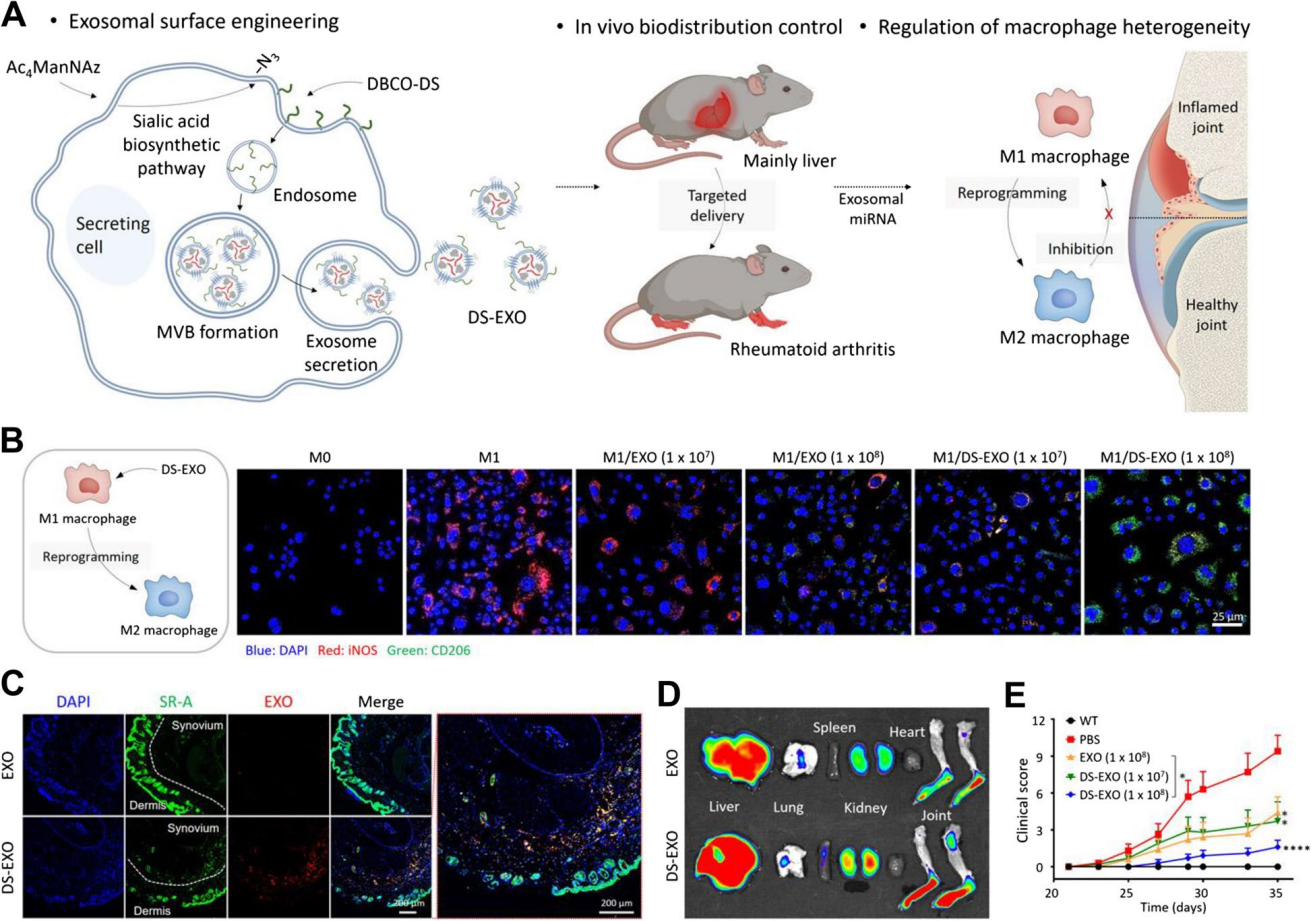
**A** Schematic illustration of metabolically engineered exosomes and mechanism regulating the macrophages in rheumatoid arthritis microenvironment (RAM). **B** Confocal microscopy images of macrophage polarization by DS-EXO. **C** Co-localization images of SR-A and EXOs in the inflamed joints of CIA mice. **D** Ex vivo organ images post-intravenous administration of the bare EXOs and DS-EXOs. **E** Clinical score of CIA mice with intravenous injection of the samples [122]. Reproduced with permission, Copyright 2021, The Author(s), Distributed under a CC-BY-NC 4.0 license (http://creativecommons.org/licenses/by-nc/4.0/), Published by American Association for the Advancement of Science

Bioorthogonal copper-free click chemistry is widely used for drug delivery, cell tracking, and tissue engineering [204, 205]. You et al. utilized this technique to obtain the surface-engineered exosomes to modulate activated macrophages (Fig. 6) [122].

The MSCs were first modified using metabolic engineering-mediated copper-free click chemistry. The Ac_4_ManNAz treated MSCs expressed the azide group on the cell surface, and were then conjugated with a dibenzocyclooctyne-conjugated dextran sulfate (DBCO-DS). Subsequently. exosomes were isolated using tangential flow filtration (TFF), which showed higher yield and consistency than conventional isolation methods, with an ultrafiltration capsule (molecular weight cut-off, MWCO = 300 kDa). The intracellular colocalization imaging of CD63 and DS during exosome biogenesis and comparison between fluorescence imaging of DS-modified exosomes and SEM images confirmed that the exosomal surface of subsequently isolated exosomes (DS-EXOs) was also modified with DS. DS on the exosomal surface facilitated their enhanced uptake into M1 macrophages and inflamed joints via SR-A, and further reprogrammed M1 macrophages to M2 phenotype. Inhibitors of miRNA, let-7b-5p, and miR-24-3p, which affect Wnt, MAPK, Hippo, and AMPK signaling pathway, suppressed M1 macrophage reprogramming to M2 phenotype, suggesting that exosomal miRNA in DS-EXO contributed to macrophage polarization. Surface engineering with DS enhanced the therapeutic efficacy of DS-EXOs in CIA by augmenting active targeting effects, implying their potential as a new cell-free therapeutic for RA.

#### Post-isolation modification

Engineering isolated exosomes facilitates exosomal surface modification with various materials ranging from proteins to polymers, to improve biodistribution or enhance targetability. Moreover, for effective delivery of small molecules, they can be loaded into exosomes in a controlled manner to avoid unexpected degradation. Several studies have generated surface-engineered exosomes, therapeutic agents-loaded exosomes, or both modified exosomes utilizing post-isolation methods to enhance their therapeutic efficacy in RA.

**Fig. 7:**
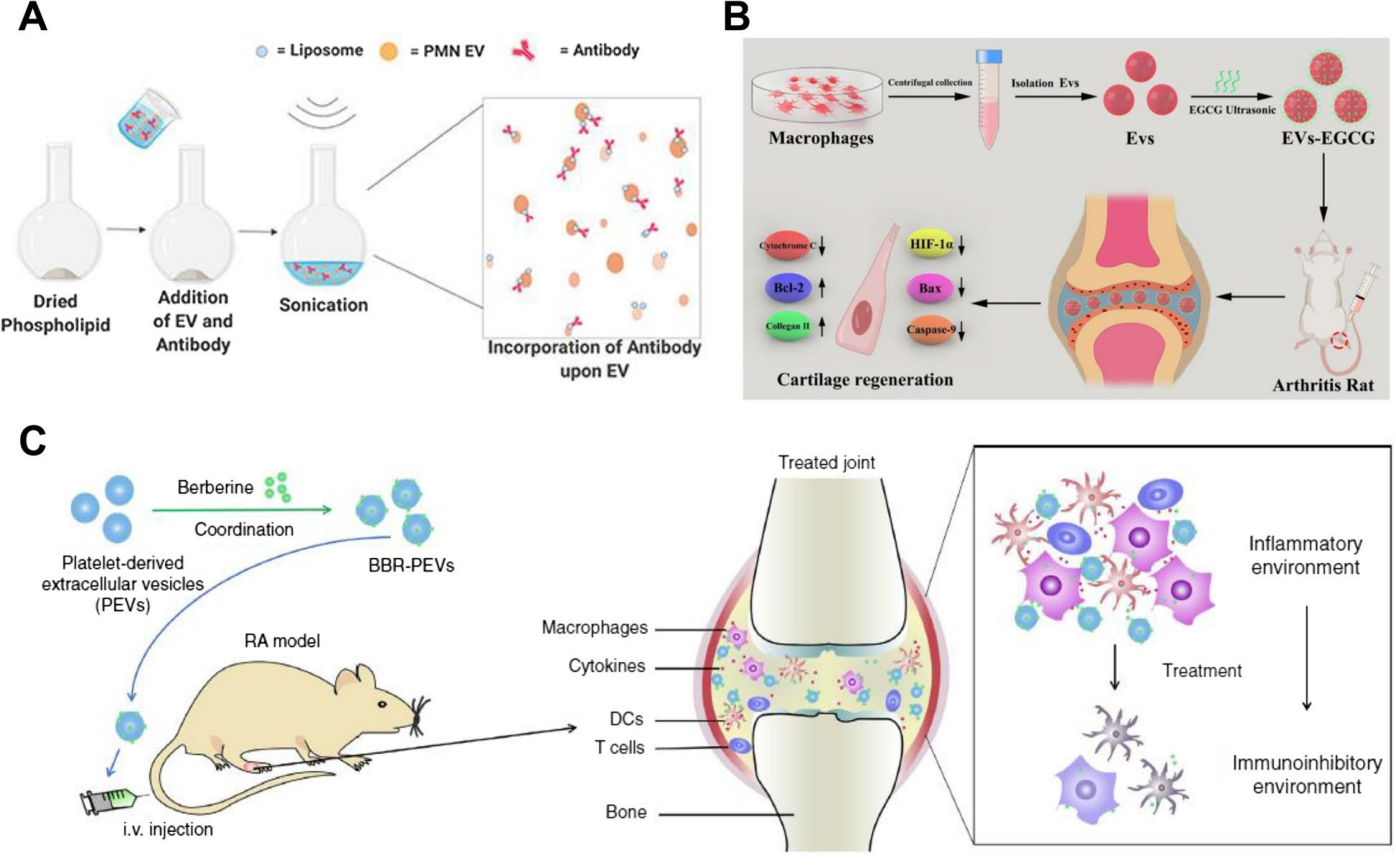
A Schematic illustration of post-isolation modification method for treatment of RA. **A** Preparation strategy of anti-ROS-CII antibodies loaded polymorphonuclear leukocytes (PMN)-derived extracellular vesicles (EVs). [170]. Reproduced with permission, Copyright 2020, Frontiers. **B** Schematic illustration of Evs-EGCG and cartilage regeneration. [171] Copyright 2021, Elsevier. **C** Schematic depicting of BBR-PEVs and RA microenvironment reprogramming [172]. Copyright 2021, Wiley

Topping et al. developed the arthritic joint targeted exosomes via incorporating inflamed cartilage-specific antibodies (Fig. 7) [170].

The polymorphonuclear leukocyte (PMNs)-derived exosomes were isolated by differential centrifugation. Then the mixture of exosomes, dried phospholipids consisting of 1,2-dioleoyl-*sn*-glycero-3-phospho-L-serine, and antibodies was sonicated for 5 min with three amplitude settings. Several kinds of antibodies, including CII post-translationally modified by oxidants-specific antibody (anti-ROS-CII), viral IL-10 fused anti-ROS-CII (anti-ROS-CII-vIL-10), anti-mouse TNFα antibody (anti-mTNF), and both anti-ROS-CII-vIL-10/anti-mTNF (anti-ROS-CII&mTNF/vIL-10), were enriched in exosomes. The data showed that the anti-ROS-CII incorporated exosomes (EV/anti-ROS-CII) bound explicitly to articular cartilage and were highly retained in the arthritic joints without off-target effects compared to non-modified exosomes. Moreover, the binding ability to both ROS-CII and mTNF of anti-ROS-CII&mTNF/vIL-10 incorporated exosomes revealed that the sonication led to incorporation of more than one antibody. In AIA, the EV/anti-ROS-CII&mTNF/vIL-10, which accumulated in the inflamed knee joints after intravenous injection, attenuated the synovial inflammation by downregulating the genes involved in pro-inflammatory response and cartilage degradation, and upregulating the genes related to anti-inflammatory response and cartilage regeneration. Furthermore, histological analysis revealed that EV/anti-ROS-CII bound to the intermediated and deep cartilage showed chondroprotection effects correlating to the genomic response.

The sonication method allows the small-sized therapeutic molecules to be loaded into exosomes. Song et al. encapsulated the epigallocatechin gallate (EGCG), a small antioxidant molecule, into macrophage-derived exosomes to improve its utility against chondrocytes in physiological conditions (Fig. 7B) [171]. The macrophage-derived exosomes were isolated from RAW cells and subsequently EGCG-loaded exosomes (EVs-EGCG) were obtained. Encapsulation and sustained-release behavior of EGCG were verified using UV–Vis absorbance. The results showed that EVs-EGCG internalized into chondrocytes, attenuated hypoxia-inducible factor 1-alpha (HIF-1α)-mediated inhibition of apoptosis, and augmented type II collagen expression, thereby alleviating RA symptoms in a rat model of CIA.

The encapsulation of the small organic compounds can be made feasible using the incubation method [172, 173]. Ma et al. developed berberine, which is known to suppress the activated macrophages and DCs, -coordinated platelet-derived exosomes (BBR-PEVs) (Fig. 7C) [172]. The PEVs were isolated by centrifugation using ultrafiltration tubes (3 kD), and the incubation of DMSO-dissolved BBR was followed. The high-performance liquid chromatography (HPLC) data showed that the loading efficiency was 6.76% and that 91.9% of BBR was sustained-released within 48 h. The BBR-PEVs selectively accumulated into inflamed joints and reshaped RA microenvironments by reducing the proportion of T cells, macrophages, and DCs, downregulating the activated phenotypes of those cells, and regulating the cytokines to a normal state; these effects collectively resulted in relief of RA symptoms. He et al. loaded curcumin (Curc) into MSC-exosomes (Curc-Exos) using the incubation method [173]. The MSC-exosomes were isolated using Exocib kit, then and mixed with Curc solution. The incubated mixtures were centrifuged by sucrose gradient, and 45 and 60% layers were selected as Curc-Exos with 18 ± 5% loading capacity. The Curc-Exos exhibited acidic pH-triggered faster release, followed by apoptosis of HIG-82 cells, a synovial membrane cell line, and downregulated pro-inflammatory mRNAs, implying their anti-inflammatory effects on FLSs.

Yan et al. utilized macrophage-derived exosomes as targeted drug delivery carriers [174]. They encapsulated dexamethasone sodium phosphate (Dex) into exosomes and modified the exosomal surface with folic acid-polyethylene glycol-cholesterol (FA-PEG-Chol) for active targeting of activated macrophages. The exosomes were isolated by differential centrifugation, and Dex was loaded (wt ratio of Exo: Dex = 1:3) using electroporation in the presence of 80 nM of trehalose to prevent aggregation. The electroporation was performed in a double poring pulse with 200 V for 5 ms and a transfer pulse of five pulses with 20 V for 50 ms at room temperature, followed by incubation in 37 ℃ PBS for 1 h to restore the exosomal membrane. Furthermore, the pre-synthesized FA-PEG-Chol was modified by post-insertion since because the cholesterol could be inserted into the lipid bilayer of the exosome. Exo/Dex and FA-PEG-Chol were mixed (wt ratio 1:5) and incubated at 37 ℃ for 2 h. The HPLC data showed the drug loading efficiency of Exo/Dex and FPC-Exo/Dex was 22.38% and 18.81%, respectively. The release of Dex was triggered by acidic pH, and the cellular uptake into activated macrophages was enhanced after surface modification, suggesting FA-mediated active targeting. In the CIA model, augmented accumulation of the FPC-Exo/Dex in inflamed joints suppressed inflammatory cell infiltration, pro-inflammatory cytokine release, as well as increase in anti-inflammatory cytokine level, which resulted in the alleviation of RA symptoms and mitigated bone erosion. The authors noted that FPC-Exo/Dex hardly manifested hepatotoxicity, suggesting their biocompatibility.

Li et al. decorated the surface of M2 macrophage-derived exosomes (EXs) with a polyarginine peptide (R9) [178]. The developed exosome was considered for potential treatment of RA. The engineered exosome exhibited higher cellular uptake and showed an excellent targeting effect. For example, the engineered exosomes showed 176% accumulation at the site of inflammation. This was due to higher Cur@EXs-R9 capture and uptake by inflammatory cells. In addition, the M2 macrophage-derived exosome effectively repolarized the macrophage at the inflamed site, and the authors proposed that Cur@EXs-R9 is a potential treatment option for RA.

#### Others

Engineering strategies utilizing both pre-isolation and post-isolation modification have been introduced to confer multifunctionality on exosomes and maximize the therapeutic effects in RA. The parent cells are first engineered to exert immunosuppressive effects, and exosomes are isolated from these engineered cells and subjected to either exosomal surface modification or cargo loading.

**Fig. 8:**
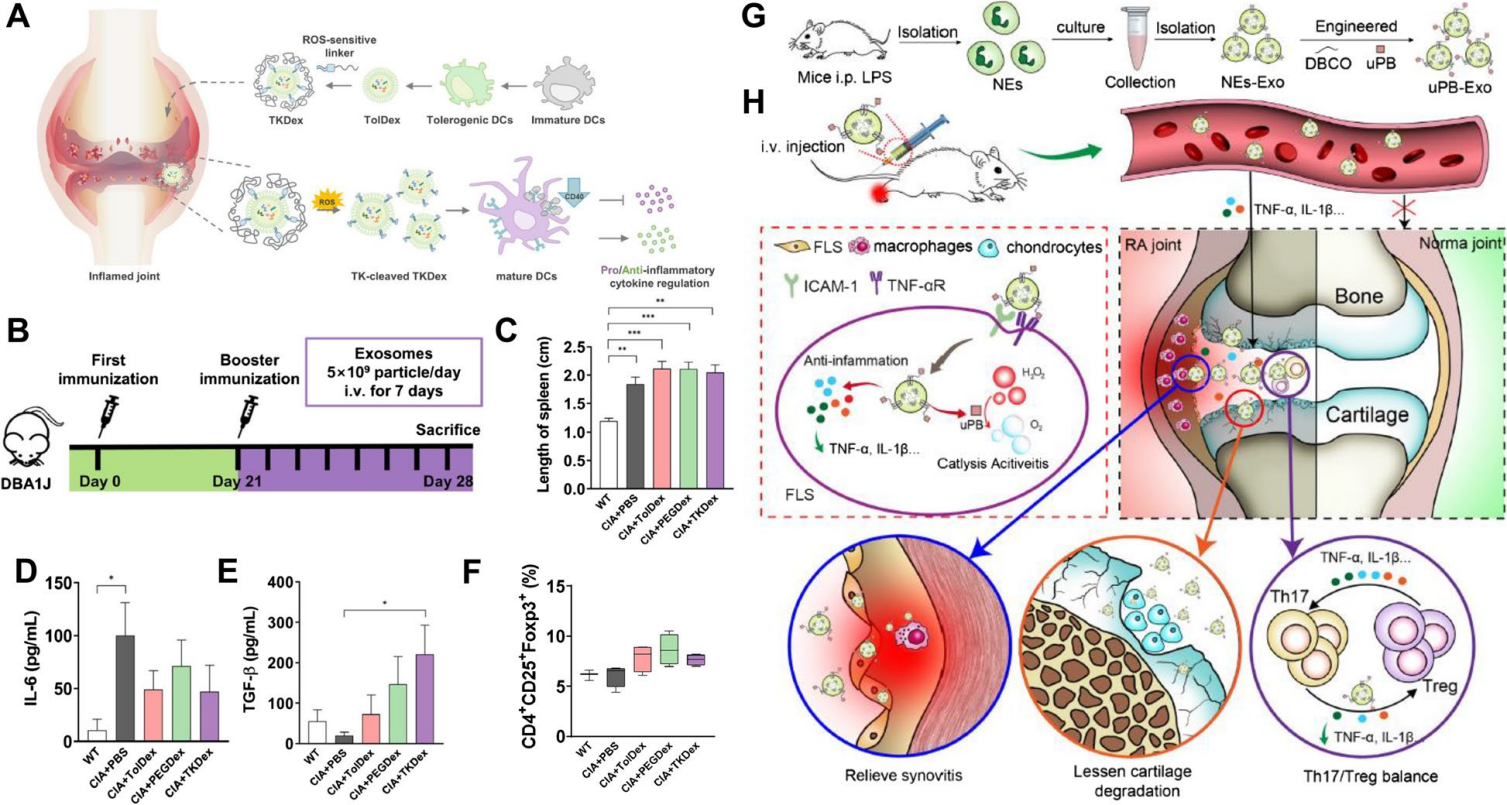
**A** Schematic illustration of TKDex and cytokine regulation. **B** Therapeutic regime of RA treatment by TKDex. **C** The size of spleen after treatment. **D**, **E** Represent cytokine level in blood. **F** Quantification of regulatory T cell after treatment of TKDex [153]. Reproduced with permission, Copyright 2021, Elsevier. **G** Schematic illustration of neutrophil-derived exosomes (NEs-Exo). **H** Illustration for therapeutic regime [175]. Reproduced with permission, Copyright 2021, Elsevier

Lee et al. developed a reactive oxygen species (ROS), one of the RAM stimuli,-responsive tolerogenic dendritic cell-derived exosomes (TKDex) (Fig. 8A) [153].

They hypothesized that PEG containing ROS-responsive moieties on the exosomal surface protected the exosomes in the bloodstream, while facilitating the exposure of exosomes in the inflamed joint, leading to their uptake into DCs, macrophages, or T cells in the RAM to induce the immunomodulation. The authors first induced immunosuppressive tolerogenic dendritic cells (TolDCs) via preconditioning DCs 2.4 cells with 40 ng/mL IL-10 for 24 h. Subsequently, the dendritic cell-derived exosomes (TolDex) were isolated utilizing TFF systems equipped with an ultrafiltration filter capsule (MWCO = 300 kDa). Meanwhile, the ROS-sensitive linkage (DSPE-TK-mPEG) for exosomal surface modification was synthesized by conjugation of thioketal (TK) linker, methoxy PEG, and 1,2-distearoyl-sn-glycero-phosphoethanolamine (DSPE). Following TolDex isolation, the DSPE-TK-mPEG was modified on the surface of TolDex via the hydrophobic insertion method. The hydrophobic insertion was performed by incubating DMSO-dissolved DSPE-TK-mPEG with TolDex. TKDex was internalized into mature DCs (mDCs) activated with LPS, whereas TolDex modified with PEG without TK (PEGDex) showed insignificant cellular uptake, suggesting its stimuli-sensitive behaviors. The in vitro data exhibited that pro-inflammatory factors were downregulated in mDCs and T cell proliferation was suppressed. In CIA, the negligible difference in the joint-to-liver ratio of TKDex and PEGDex implied that TK moiety did not contribute to former’s degree of accumulation in the lesions. Decrease in IL-6 level, increased TGF-β level, and increased proportion of Tregs suggested that TKDex could modulate T cells as well as DCs in inflamed joints. It is noteworthy that the therapeutic activity of Dex are induced in only ROS-overproduced RAM.

Zhang et al. modified ultrasmall Prussian blue nanoparticles (uPB), nanoenzyme, on the surface of neutrophil-derived exosomes to regulate the RAM (Fig. 8G) [175]. Since LFA-1 on the activated neutrophil membrane can bind with activated FLSs, chondrocytes, and macrophages via intracellular activation molecule 1 (ICAM-1), LPS was injected into mice, followed by the isolation of neutrophils. Subsequently, the neutrophil-derived exosomes (NEs-Exo), possessing the LFA-1, were isolated by differential centrifugation. In addition, an antioxidative enzyme mimic, Prussian blue was fabricated into ultrasmall nanoparticles and further anchored to the exosomal surface to relieve the ROS-abundant conditions in inflamed joints. DBCO-sulfo-NHS was conjugated on the exosomal surface in advance, and N_3_-uPB was linked by copper-free click chemistry. The uPB-Exo showed enhanced uptake into activated FLSs, chondrocytes, and macrophages, as well as ROS scavenging in vitro, indicating the role of LFA-1 in active targeting and activity of nanozymes, respectively. Moreover, uPB-Exo revealed protective effects against oxidative stress and cytokines secreted from apoptotic cells by mimicking SOD-2 and inhibiting apoptosis of activated FLSs, chondrocytes, and macrophages. The authors also validated that apoptosis was related PI3K/AKT signaling pathway. In CIA, both NEs-Exo and uPB-Exo were highly accumulated into paws and penetrated deep into the cartilage. The significant differences in the reduction of TNF-α, IL-1β, and Th1, as well as the increase in Treg count suggested the synergistic effects of uPB with NEs-Exo.

**Fig. 9:**
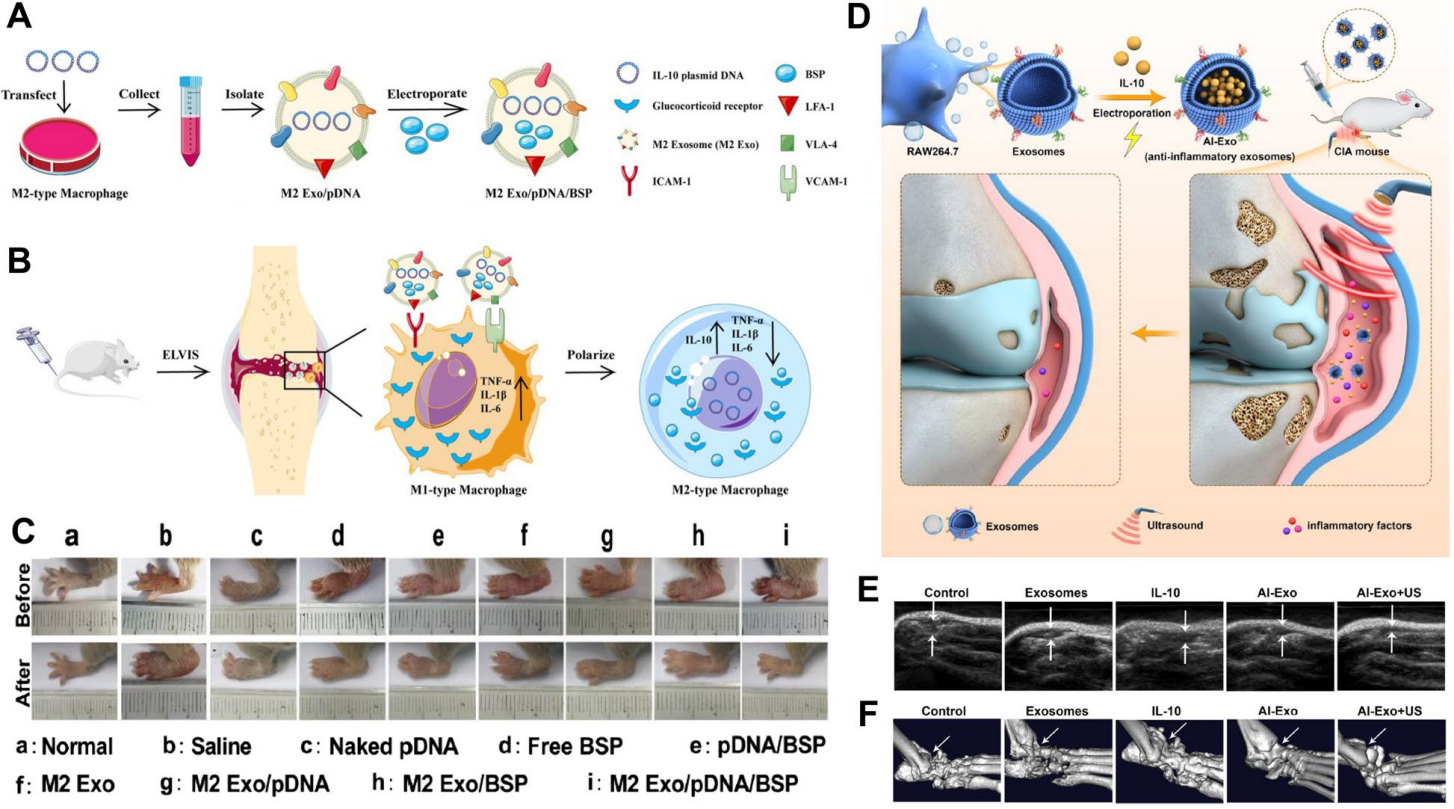
**A** Schematic illustration of M2-macrophage derived exosomes (M2-Exo/pDNA). **B** Therapeutic regime of M2-Exo/pDNA and macrophage polarization. **C** Photographs of paw thickness [176]. Reproduced with permission, Copyright 2022, Elsevier. **D** Schematic illustration of anti-inflammatory exosomes (AI-Exo). **E** Photoacoustic images of joint. **F** skeletal structure of paw joint [177]. Reproduced with permission, Copyright 2022, Royal Society of Chemistry

M2 macrophage-derived exosomes loaded with a plasmid DNA encoding IL-10 (IL-10 pDNA) and synthetic glucocorticoid were developed for macrophage re-polarization in RA (Fig. 9A) [176].

The authors aimed to inhibit pro-inflammatory factors and promote M1 polarization to M2 macrophages. M2 macrophages (RAW 264.7 cells) were induced with 100 ng/mL IL-4. The pDNA-lipid complexes, comprising earlier synthesized IL-10 pDNA and transfection agent, were incubated with M2 macrophages at 37 ℃ for 3 days. Subsequently, the IL-10 pDNA encapsulated M2-derived exosomes (M2 Exo/pDNA) were isolated using differential centrifugation with ultrafiltration. In brief, supernatant centrifuged at 300 g, 2000 g, and 10,000 g was concentrated using ultrafiltration through MWCO 100 kDa filter, followed by ultracentrifugation at 120,000 g. Lastly, to obtain the betamethasone sodium phosphate (BSP) loaded M2 Exo/pDNA (M2 Exo/pDNA/BSP), the M2 Exo/pDNA and BSP were mixed (wt ratio 1:2) with 80 nM trehalose and electroporated with poring pulse at 200 V, pulsed twice for 5 ms and transfer pulse for five pulses at 20 V for 50 ms at room temperature. M2 Exo/pDNA/BSP showed sustained release of BSP (approximately 98.93% within 20 h). The enhanced uptake of M2 Exo into activated macrophages was attributed to the overexpression of LFA-1 or VLA-4 on M2 Exo, which can bind to ICAM-1 or VCAM-1 on activated macrophages, suggesting the inherent targetability of M2 Exo to M1 macrophages. As expected, the M2 Exo/pDNA/BSP exhibited maximal effects on M1 to M2 polarization compared to free BSP, pDNA/BSP, M2 Exo, M2 Exo/pDNA, and M2 Exo/BSP. In CIA, highly accumulated M2 Exo/pDNA/BSP in inflamed joints regulated pro-and anti-inflammatory cytokines and protected the cartilage, without inducing hepatotoxicity; Thus, this system led to effective co-delivery of genes and drugs without side effects allowing synergy.

Tang et al. utilized an external energy source to improve the therapeutic effects in RA (Fig. 9D) [177]. The authors prepared anti-inflammatory exosomes (AI-Exo) by loading IL-10 into macrophage (M0 type)-derived exosomes and irradiated using non-invasive ultrasound (US) during therapy to enhance blood vessel permeability to accelerate drug uptake into tissues and cells. The AI-Exo was prepared as follows; 1) Exosomes were isolated from RAW 264.7 cells using ExoQuick-TC®ULTRA EV Isolation kit, and 2) IL-10 and exosomes were electroporated (wt ratio 2:15, 600 V for 90 μs, 5 times pulses with 1 s interval). The in vitro data showed that US irradiation enhanced the cellular uptake of AI-Exo into activated macrophages. Similarly, the US augmented the accumulation of AI-Exo into inflamed joints in CIA, alleviating RA symptoms. The mechanistic analysis verified that the therapeutic effect of AI-Exo^+^US group was attributed to the reduction in IL-6 and TNF-α level and decrease CD86/CD206 ratio in macrophages. The authors noted that IL-10 plays a role in promoting polarization of M1 macrophages to M2 macrophages, and US can enhance the targetability by promoting the activated macrophage phagocytosis of AI-Exo.

Engineering other subpopulations of extracellular vesicles, including matrix-bound nanovesicles (MBV) and MV, was also reported. The MBVs are similar to MV; however, they are anchored to the extracellular matrix (ECM) and identified as functional components of ECM [206]. Crum et al. verified that the MBV isolated from urinary bladder ECM by enzymatic digestion attenuated the inflammation in pristane-induced arthritis via synovial and splenic macrophage polarization from M1 to M2 phenotype [207]. Meanwhile, MV was used for developing biomimetic nanoparticles. The zeolitic imidazolate framework-8 (ZIF-8), one of the common metal–organic frameworks (MOF), encapsulated the MTX and was camouflaged with macrophage-derived MV and further modified with DSPE-PEG-FA by hydrophobic insertion to enhance the biocompatibility and targetability [208]. In the same group, the Dex-encapsulated and macrophage-derived MV-coated ZIF-8 was also developed for targeted delivery to arthritic joints [209]. The macrophage-derived MV was employed to coat the poly(lactic-*co*-glycolic acid) (PLGA) nanoparticles, eliciting enhanced RA targeting [210].

## Challenges and perspectives

Considering the biological effects of the exosome in cell-to-cell communication, using an exosome as an RA therapeutics is a potential approach for treating RA. In addition to DC and T cells, exosomes derived from various cells, such as NK cells, can enhance each cell's function and modulate the disease microenvironment. Although the etiology of RA has not been precisely elucidated, therapeutic strategies that convert autoimmunity into a state of immune tolerance have been proposed. Because systemic immunosuppression induces undesirable immunodeficiency, the importance of inducing antigen-specific tolerance is emerging to treat autoimmune diseases effectively [211]. Recently, OA or chronic multiple sclerosis was reportedly treated in an antigen-specific manner by inducing tolerogenic DC using nanovaccines utilizing antigen-specificity [212, 213]. Although Rheumavax, an autologous DC modified by exposure to citrullinated peptides, was introduced several years ago and was safe and effective in clinical trials with a single administration, issues related to cell-based therapy remain a challenge. In this perspective, exosomes are non-immunogenic and less toxic than organic/inorganic therapeutic nanoparticles; therefore, they can be used to substitute cell-based therapy. Moreover, DC-derived exosomes can be developed into tolerogenic nanovaccines through various engineering methods. Development of antigen-specific nanovaccines using DC exosomes is a promising strategy for developing novel RA therapeutics, thereby preventing non-specific immune system downregulation.

Challenges such as low in vivo stability and unspecific uptake of bare exosomes have been identified, the need to utilize engineered exosomes has emerged. Engineered exosomes equipped with targeting moieties or PEGylation for long-term circulation have emerged as excellent alternatives as they overcome low in vivo biostability of bare exosomes.

However, considering that mass production of engineered exosomes is challenging, the issues related to their producibility and cost need to be addressed. Some strategies, such as three-dimensional cultivation using hallow-fiber bioreactor [214], and focused US irradiation [215], can improve the production efficiency; however, other challenges remain. In addition, the exosomes have a complex structure compared to conventional drugs. Therefore, difficulties in their characterization required for drug approval hinders further application of engineered exosomes in clinical settings. For example, problems of animal serum retention in the cell culture medium used for exosome production can be a huddle for the clinical application of exosomes, as well as the limitation of the human application of stem cells.

Exosome-based therapies, encompassing bare and engineered exosomes, offer promising prospects for RA treatment. Even though both strategies have been studied with various types of exosomes, it is crucial to weigh the potential advantages against the limitation of each approach. Bare exosomes possess inherent immunomodulatory properties depending on the origin cell types, and autologous or allogeneic exosomes are biocompatible and have lower immunogenicity, minimizing the risk of immune responses or adverse reactions. Nevertheless, the variability of naturally controlled cargoes might lead to inconsistent therapeutic efficacy. In addition, since the mode of action of bare exosomes' immunomodulatory effects and anti-inflammatory effects are complex and unpredictable, the diverse exosomal content and interaction with various types of cells might lead to undesirable biological effects.

On the other hand, various therapeutic cargo like anti-inflammatory drugs, disease-modifying molecules, and immunomodulatory molecules can be loaded within engineered exosomes, and the loading efficiency can be precisely controlled. The targeting moieties on exosomal surfaces enable exosome delivery at the intended site of action, resulting in enhanced therapeutic efficacy and lower side effects. Furthermore, the engineered exosomes can be personalized by customizing to the needs of individual patients based on the heterogeneity of specific diseases. Since the engineered exosomes have more chance to recognize immune systems than bare exosomes, the engineering strategy should augment the biocompatibility for lowing the immunogenicity.

In addition to using the exosome directly, in situ exosome biogenesis is an emerging strategy. In situ exosome biogenesis modulation is a strategy without the production process of the exosome, which is expensive. For example, the strategy to inhibit the secretion of exosomes derived from the cell population that overproduces exosomes in the disease microenvironment showed promising microenvironment modulation capability in the pre-clinical state. Conversely, strategies to induce the secretion of exosomes from suppressed cell populations within the disease microenvironment can be considered. Because exosomes function as cell avatars, increased secretion of exosomes can be utilized to restore, reinvigorate, or suppress biological functions within the microenvironment of the suppressor cell population.

## Conclusion

In conclusion, using exosomes as a potential therapeutic approach for RA is promising due to their non-immunogenicity and less toxicity than other therapeutic nanoparticles. However, issues related to the production and characterization of engineered exosomes need to be addressed. Therefore, further research is required to address these challenges and fully utilize the potential of exosomes in treating RA and other autoimmune diseases.

## Data Availability

Not applicable.

## Abbreviations

AA: Ascorbic acid
Ac_4_ManNAz: N-azidoacetyl-D-mannosamine
AIA: Antigen-induced arthritis
AnxA1: Annexin A1
APC: Antigen-presenting cells
BLI: Biolayer interferometry
BMDM: Bone marrow-derived macrophages
BMEVs: Bovine milk-derived exosomes
BSP: Betamethasone sodium phosphate
CDT: Chemotherapy
Ce6: Chlorin e6
Chol: Cholesterol
CIA: Collagen-induced arthritis
CRISPR: Clustered regularly interspaced short palindromic repeats
CTLA-4 Ig: Cytotoxic T lymphocyte-associated antigen-4-Ig
Curc: Curcumin
CXCL9: C-X-C motif chemokine ligand 9
DCs: Dendritic cells
Dex: Dendritic cells-derived exosomes
DiR: 1,1-Dioctadecyl-3,3,3,3-tetramethylindotricarbocyaine iodide
DMARDs: Disease-modifying anti-rheumatic drugs
DOX: Doxorubicin
DS: Dextran sulfate
DSPE: 1,2-Distearoyl-sn-glycero-phosphoethanolamine
DTH: Delayed hypersensitivity
ECs: Endothelial cells
EGCG: Epigallocatechin gallate
EGFR: Epidermal growth factor receptor
ESCRT: Endosomal sorting complex responsible for transport
EVs: Extracellular vesicles
FA: Folic acid
FLSs: Firoblast-like synoviocytes
GEO: Gene expression omnibus
GMPs: Good manufacturing practices
GMSCs: Gingical mesenchymal stem cells
HA: Hyaluronic acid
HCQ: Hydroxychloroquine
HLA: Human leukocyte antigen
HPLC: High-performance liquid chromatography
hUCMSCs: Human umbilical cord mesenchymal stem cells
ICAM-1: Intracellular activation molecule 1
ICG: Indocyanine green
IDO: Indoleamine-2,3-dioxygenase
iNOS: Nitric oxide synthase
JAK: Janus kinase
LPS: Lipopolysaccharide
MAPK: Mitogen-activated protein kinase
MB: Molecular beacon
MMPs: Matrix metalloproteinases
MSCs: Mesenchymal stem cells
MVBs: Multivesicular bodies
MVs: Microvesicles
NF-κB: Nuclear factor kappa B
NO: Nitric oxide
NSAIDs: Nonsteroidal anti-inflammatory drugs
PDA: Polydopamine
PDT: Photodynamic therapy
PEG: Poly(ethylene glycol)
PET: Positron emission tomography
PEVs: Platelet-derived exosomes
PGE2: Prostaglandin E2
PLGA: poly(lactic-*co*-glycolic acid)
PMNs: Polymorphonuclear leukocyte
PTX: Paclitaxel
RA: Rheumatoid arthritis
RAM: Rheumatoid arthritis microenvironment
RBC: Red blood cells
RNP: Ribonucleoprotein
ROS: Reactive oxygen species
SBC: Sodium bicarbonate
SCI: Spinal cord injury
SDT: Sonodynamic therapy
sgRNA: Single guide RNA
SR-A: Scavenger receptor class A
STAT3: Signal transducers and activates transcription 3
TALENs: Transcription activator-like effector nucleases
TFF: Tangential flow filtration
Th: T-helper cells
TK: Thioketal
TNF-α: Tumor necrosis factor-α
TPP: Triphenylphosphonium
Tr1: T regulatory type 1 cells
Tregs: Regulatory T cells
US: Ultrasound
VEGF: Vascular endothelial growth factor
ZFNs: Zinc finger nucleases
ZnS: Zinc sulfide
